# Primary healthcare professionals’ knowledge and self-efficacy on palliative care

**DOI:** 10.1590/0034-7167-2024-0056

**Published:** 2025-11-28

**Authors:** Elisa Cavalheiro Libardi, Beatriz Aparecida Ozello Gutierrez

**Affiliations:** I Universidade de São Paulo. São Paulo, SP, Brazil

**Keywords:** Palliative Care, Knowledge, Self-Efficacy, Primary Health Care, Multiprofessional Team., Cuidados Paliativos, Conocimiento, Autoeficacia, Atención Primaria de Salud, Equipo Multiprofesional.

## Abstract

**Objective:**

to obtain information regarding knowledge and self-efficacy about palliative care among professionals working in primary healthcare.

**Methods:**

a total of 146 professionals from different professions were assessed through a sociodemographic and professional questionnaire and the Questionário de Conhecimento e Autoeficácia Sobre Cuidados Paliativos (BPW-BR).

**Results:**

there are significant deficits in knowledge and self-efficacy among all primary healthcare professionals about palliative care. Theoretical and practical knowledge, communication skills, pharmacological and non-pharmacological pain management, and better integration of knowledge among primary healthcare teams about palliative care are needed.

**Conclusions:**

the study showed an overview of the knowledge and self-efficacy regarding palliative care among professionals who make up primary healthcare teams, but it needs to be expanded in number, variety of participants and regional scope, for results that express the Brazilian reality.

## RESUMEN

**Objetivo:**

recopilar información sobre el conocimiento y la autoeficacia en cuidados paliativos entre profesionales de Atención Primaria de Salud.

**Métodos:**

se evaluó a 146 profesionales de diferentes profesiones mediante un cuestionario sociodemográfico y profesional, así como el Questionário de Conhecimento e Autoeficácia Sobre Cuidados Paliativos (BPW-BR).

**Resultados:**

existen déficits significativos de conocimiento y autoeficacia en cuidados paliativos entre todos los profesionales de Atención Primaria. Se requieren conocimientos teóricos y prácticos, habilidades de comunicación, manejo del dolor farmacológico y no farmacológico, y una mejor integración de los conocimientos sobre cuidados paliativos entre los equipos de Atención Primaria.

**Conclusiones:**

el estudio mostró una visión general del conocimiento y autoeficacia acerca de los cuidados paliativos entre los profesionales que integran equipos de Atención Primaria de Salud, pero necesita ser ampliado en número, variedad de participantes y alcance regional, para resultados que expresen la realidad brasileña.

## INTRODUCTION

According to the organization of healthcare levels in the Brazilian Health System, primary healthcare (PHC) is responsible for coordinating and organizing care, and is the main gateway for users to enter the health system. PHC is responsible for resolving most health problems, referring users to other levels of care only in more complex situations or situations that require technological resources not available at this level of care^(1)^.

In recent years, palliative care (PC) has become part of the services offered by PHC^(2)^. PC consists of a set of interventions aimed at individuals of any age group and their families affected by life-threatening diseases. Its main objective is to alleviate suffering, and is indicated for people diagnosed with progressive chronic diseases and an unfavorable prognosis, which generate physical, emotional, social and spiritual suffering for both patients and their caregivers and support network^(3)^.

The inclusion of PC in PHC is a promising strategy for early identification and longitudinal monitoring of eligible patients from the moment of diagnosis. In addition, it favors geographical, emotional and cultural proximity between health teams, patients and their families, facilitating maintenance of care at home^(4)^.

Despite the increased demand for palliative care, Brazil still has an unsatisfactory performance on the international stage, with an insufficient number of specialized services^(5)^ and suboptimal pain control, compromising quality of life and death^(6)^. However, strengthening initiatives have been implemented, with emphasis on the establishment of the Brazilian National Palliative Care Policy (In Portuguese, *Política Nacional de Cuidados Paliativos* - PNCP) in May 2024. This policy aims to increase funding in all areas of management, train healthcare professionals, expand specialized interdisciplinary teams, promote education of the population about palliative care and ensure its implementation throughout the Healthcare Network (In Portuguese, *Rede de Atenção à Saúde* - RAS)^(7)^.

Quality of care in PC is directly related to interprofessional and interdisciplinary teams’ performance, technically and scientifically trained, with clinical skills that contemplate the multiple suffering dimensions - physical, emotional, spiritual and social - of patients and their support networks^(8)^. PHC, due to its organizational characteristics, is an environment conducive to these teams’ work, as long as professionals have adequate knowledge on the subject^(8)^.

International studies demonstrate unsatisfactory levels of knowledge and self-efficacy in PC among PHC professionals, both in developed and developing countries. In Spain, for instance, an assessment carried out with 561 PHC physicians and nurses revealed that 65.5% of nurses and 32.5% of physicians had weak or insufficient knowledge. However, professionals who had participated in continuing education, specialization courses or who had contact with PC during their undergraduate studies showed significantly higher performance^(9)^.

In Malaysia, a country where PC has also recently been implemented in PHC, a study with 271 physicians found that only 12.6% had received some training, even if specific, on the subject. The results showed a positive correlation between greater knowledge and more appropriate behaviors in PC^(10)^, reinforcing the importance of research that identifies difficulties and potentialities in PHC professionals’ knowledge, with a view to building more effective training strategies.

Self-efficacy in PC is also a fundamental aspect, as it directly influences the quality of care provided^(11)^. Although still little explored, this dimension reveals relevant data. A study carried out in the southern United States of America showed that nurses with greater self-efficacy in PC feel more prepared to discuss the dying process, offer support to patients and family members, and manage symptoms such as delirium, pain and respiratory changes^(11)^. Low self-efficacy, in turn, compromises the planning of actions and identification of patients eligible for PC^(12)^.

In Brazil, there is still a shortage of studies that systematically investigate PHC professionals’ knowledge and self-efficacy in relation to PC - a worrying gap, given the growth of the area and the need for continuous training. Research conducted in Minas Gerais revealed that PHC nurses have limited knowledge, especially regarding the definition and philosophical principles of PC, highlighting the urgency of investments in professional training^(13)^.

In addition to the small number of studies, there is also a limited diversity in the professional categories investigated. The research focuses mainly on physicians and nurses, with few studies including social workers and no studies covering other members of multidisciplinary teams^(14)^.

Given this scenario, there is a need to expand research on knowledge and self-efficacy in PC among PHC professionals, considering the diversity of training. This will allow the formulation of more assertive educational strategies, favoring the qualification of palliative care at this level of healthcare.

## OBJECTIVES

General objective: to assess the level of knowledge and self-efficacy in PC of the multidisciplinary team that makes up the PHC network in the city of São Paulo.

Specific objective: to identify, through the responses to the *Questionário de Conhecimento e Autoeficácia sobre Cuidados Paliativos* (BPW-BR), the points to be improved in the practice of healthcare professionals with higher education working in PHC units in the city of São Paulo, considering the domains of pain, symptom control, general knowledge on the subject and attitudes towards death.

## METHODS

### Ethical aspects

The study was conducted in accordance with national and international ethics guidelines, identified by the Certificate of Presentation for Ethical Consideration 52538721.3.3001.0086, and was approved by the Research Ethics Committees of the São Paulo Municipal Health Department and the *Universidade de São Paulo* School of Arts, Sciences and Humanities, under Opinion 5.096.355, which is attached to this submission.

All participants were previously informed about the procedures to be performed and instructed to complete the Informed Consent Form (ICF), which enabled the collection of data for research and publication. Professionals received a copy of the document by email, automatically, after completing the ICF.

### Study design, period and setting

This is an exploratory cross-sectional observational study with quantitative data analysis, with an assessment of healthcare professionals who worked in units linked to the PHC of the southeast, east and north health regions of the city of São Paulo, SP, Brazil, in Basic Health Units (BHU) and Basic Health Units with integrated Outpatient Medical Care (BHU/OMC integrated). The data collection period was between February 2022 and December 2022.

To construct the study, the Consensus Reporting Items for Studies in Primary Care-CRISP Statement was used, which aims to guide research carried out in PHC^(15)^.

### Sample and inclusion and exclusion criteria

The sample consisted of 146 healthcare professionals with higher education, linked to PHC, working in the Family Health Strategy (FHS), Family Health Support Center (In Portuguese, *Núcleo de Apoio à Saúde da Família* - NASF), Multidisciplinary Home Care Team (MHCT) and Multidisciplinary Support Team (MST) programs. Only professionals who agreed to participate in the research were included. Those who, at the time of data collection, did not have enough time to respond to the instrument, were away on sick leave or vacation, or who consulted other people or informational materials while completing the questionnaire were excluded.

### Study protocol

Data collection was carried out using an electronic form (Google Forms^®^), containing two instruments: a sociodemographic and professional characterization questionnaire and BPW-BR.

The sociodemographic questionnaire included information on age, sex, religion and marital status. Professional variables included type and time since graduation, graduate studies, time working in the unit, previous experience with PC and participation in courses or training on the subject.

BPW-BR consists of 38 items, 23 of which are intended to assess professionals’ knowledge about PC, addressing topics such as pain management, symptom control, general knowledge and attitudes towards death, and 15 items aimed at analyzing self-efficacy in providing PC. The items are presented on a four-point Likert scale: 4 - “true”; 3 - “more or less true”; 2 - “hardly true”; and 1 - “not true”^1 6^. Originally developed in Germany^(16)^, BPW-BR was adapted and validated for the PHC context in Brazil with professionals from different health areas^(17)^ (Chart 1).

**Chart 1:** *Questionário de Conhecimento e Autoeficácia sobre Cuidados Paliativos* (BPW-BR) questions

*Questionário de Conhecimento e Autoeficácia sobre Cuidados Paliativos (BPW-BR)*	
*Escala de Likert: Verdadeiro=4; Mais ou menos verdadeiro=3; Dificilmente verdadeiro=2; Não é verdadeiro=1*	
***C1-*** *Cuidados Paliativos nunca devem ser combinados com tratamentos curativos.*	***RE=1***
***C2 -*** *Medicamentos anti-inflamatórios não esteróides devem ser excluídos durante o uso esporádico de opióides.*	***RE=1***
***C3 -*** *Administração de fluidos por via subcutânea (hipodermóclise) é necessária para aliviar a boca seca (xerostomia) em pacientes em final de vida.*	***RE=4***
***C4 -*** *No caso do paciente com dores, que está na fase final de vida, é adequado utilizar um adesivo transdérmico.*	***RE=4***
***C5 -*** *As terapias complementares são importantes para o controle da dor.*	***RE=4***
***C6 -*** *É sempre importante que os membros da família fiquem com o paciente até que ocorra a morte.*	***RE=4***
***C7 -*** *A constipação deve ser aceita como um sintoma secundário, pois a redução da dor é mais importante.*	***RE=4***
***C8 -*** *Os cuidados paliativos requerem apoio emocional constante.*	***RE=4***
***C9 -*** *Na velhice, devido às inúmeras experiências de perda, as pessoas aprenderam a lidar com o luto de forma independente.*	***RE=1***
***C10 -*** *A filosofia dos cuidados paliativos significa que não são mais realizados tratamentos para prolongar a vida.*	***RE=4***
***C11 -*** *Medo e exaustão diminuem a intensidade da dor.*	***RE=1***
***C12 -*** *Pacientes com doenças que ameaçam a vida devem sempre saber da verdade para que possam se preparar para a morte.*	***RE=4***
***C13 -*** *Os membros da equipe não precisam ter fé para poder acompanhar espiritualmente pacientes em fase final de vida.*	***RE=4***
***C14 -*** *Paciente em cuidados paliativos deve aceitar a morte.*	***RE=1***
***C15 -*** *Habilidades de comunicação podem ser aprendidas.*	***RE=4***
***C16 -*** *A morte do paciente não deve ser comunicada para outros pacientes próximos dele e em situação semelhante, para evitar inquietação.*	***RE=4***
***C17 -*** *Os aspectos médicos do tratamento sempre têm prioridade nos cuidados paliativos.*	***RE=1***
***C18 -*** *Rituais visíveis e despedidas quando o paciente morre, devem ser evitados para não causar inquietação.*	***RE=1***
***C19 -*** *O uso de antidepressivos na terapia da dor não faz sentido.*	***RE=1***
***C20 -*** *Ao administrar opioides, os analgésicos adjuvantes não são necessários.*	***RE=1***
***C21 -*** *A fase final compreende os últimos três dias de vida.*	***RE=4***
***C22 -*** *Os sentimentos do cuidador (por exemplo, desprezo) podem ser expressados no acompanhamento.*	***RE=4***
***C23 -*** *As necessidades fisiológicas (por exemplo, sexualidade) ainda são importantes na fase final de vida.*	***RE=4***
***AE1 -*** *Coletar dados objetivos que descrevam o nível de dor do paciente.*	
***AE2 -*** *Orientar o paciente sobre como reduzir a náusea.*	
***AE3 -*** *Informar o paciente e os familiares sobre os cuidados paliativos na instituição.*	
***AE4 -*** *Convencer o médico de família sobre a necessidade dos cuidados paliativos.*	
***AE5 -*** *Reconhecer os problemas atuais do paciente em seu meio social e discuti-los com ele.*	
***AE6 -*** *Planejar um encaminhamento para serviços de cuidados paliativos.*	
***AE7 -*** *Conversar com o paciente ansioso e seus entes queridos para que se sintam seguros.*	
***AE8 -*** *Reconhecer e responder adequadamente às necessidades complexas do paciente em cuidados paliativos.*	
***AE9 -*** *Oferecer ao paciente com dor terapias complementares de relaxamento, adequadas para ele.*	
***AE10 -*** *Conversar com um paciente que expressa o desejo de antecipar a morte.*	
***AE11 -*** *Realizar higiene oral apropriada de pacientes em final de vida.*	
***AE12 -*** *Orientar o paciente sobre os possíveis efeitos colaterais dos medicamentos prescritos.*	
***AE13 -*** *Reconhecer problemas específicos de saúde mental nos pacientes.*	
***AE14 -*** *Integrar aspectos culturais relacionados à morte e o morrer ao cuidar de pacientes em final de vida.*	
***AE15 -*** *Ter empatia e respeitar as condições de vida desconhecidas, dinâmicas familiares e necessidades associadas dos pacientes.*	

*Note: C = conhecimento (knowledge); AE = autoeficácia (self-efficacy); RE = resposta esperada (expected answer).*

The instrument demonstrated good content validity (CVI > 0.83), median internal consistency for the “knowledge” construct (Cronbach’s alpha = 0.486) and excellent for “self-efficacy” (Cronbach’s alpha = 0.852), in addition to good overall reliability (ICC > 0.5) and excellent reproducibility (p = 0.046) ^(17)^. It is, therefore, a valid and appropriate instrument for use in studies in the context of Brazilian PHC, contributing to the identification of points to be improved in clinical practice in PC as well as supporting reflections for professional qualification and improvement of services^(17)^.

### Results analysis

As methods of analysis of results, descriptive analysis was used to characterize the sociodemographic and professional information of quantitative variables. For qualitative variables, the equality of two proportions test was applied in order to compare frequencies between groups. The Kruskal-Wallis test, a nonparametric technique, was used to compare distributions of an ordinal or continuous variable between three or more independent groups, especially when the data did not present a normal distribution. Spearman’s correlation was used to assess the relationship between two ordinal or nonparametric quantitative variables, allowing verification of the direction and strength of the association between them, even in the absence of linearity or normality.

In the interpretation of correlation coefficients, p-values ≤ 0.05 were considered indicative of statistical significance, weak (values between 0 and 0.25), regular (0.25 to 0.50), good (0.50 to 0.75) and excellent (0.75 to 1.00) correlation.

## RESULTS

Based on participant sociodemographic and professional characteristics, it was observed that professionals were between 24 and 64 years old, the majority of whom were female (83.6%), with 43.8% self-declared as married and 36.3% of Catholic religion. The mean time since graduation was 12 years, while the mean time working in PHC was eight years.

As shown in Table 1, 45.9% of participants worked in BHUs. The sample was composed of professionals from different health categories, with 43.8% being nurses, 19.2% physicians, and the rest were classified as “other professionals”, such as social workers (8.9%), pharmacists (4.8%), physiotherapists (5.5%), speech therapists (0.7%), health unit managers (2.7%), nutritionists (5.5%), dentists (6.2%), psychologists (2.1%) and occupational therapists (0.7%).

**Table 1:** Professional and academic background data of participants (N = 146)

		n	%	*p* value			n	%	*p* value
**Profession**	Social worker	13	8.9%	<0.001	**Type of institution where you graduated**	Private	114	78.1%	<0.001
	Nurse	64	43.8%	Ref.		Public	32	21.9%	
	Pharmaceutical	7	4.8%	<0.001	**Graduate studies**	No	13	8.9%	<0.001
	Physiotherapist	8	5.5%	<0.001		Yes	133	91.1%	
	Speech therapist	1	0.7%	<0.001	**Type of graduate degree**	Doctoral degree	2	1.4%	<0.001
	Manager	4	2.7%	<0.001		Specialization	129	88.4%	Ref.
	Physician	28	19.2%	<0.001		Master’s degree	2	1.4%	<0.001
	Nutritionist	8	5.5%	<0.001		There is no	13	8.9%	<0.001
	Dentist	9	6.2%	<0.001	**Training** **in PC**	No	129	88.4%	<0.001
	Psychologist	3	2.1%	<0.001		Yes	17	11.6%	
	Occupational therapist	1	0.7%	<0.001	**Contact with patients** **in PC**	No	38	26.0%	<0.001
						Yes	108	74.0%	
**Type of unit/team working in PHC**	BHU/OMC	8	5.5%	<0.001		NASF	4	2.7%	<0.001
	MST	1	0.7%	<0.001		IAP	1	0.7%	<0.001
	MHCT	30	20.5%	<0.001		BHU	67	45.9%	Ref.
	FHS	34	23.3%	<0.001					

*Note: PC = palliative care; NASF = Family Health Support Center; PHC = primary healthcare; BHU/OMC = Basic Health Units with Outpatient Medical Care; MST = Multidisciplinary Support Team; MHCT = Multiprofessional Home Care Team; FHS = Family Health Strategy; IAP = Integrated Actions Program; BHU = Basic Health Unit; N = number of participants; Ref = question with the highest number of answers.*

**Table 2:** Correlation of quantitative factors with the *Questionário de Conhecimento e Autoeficácia sobre Cuidados Paliativos* (BPW-BR) questions

	Age		Time since graduation		Job tenure		Time working in primary healthcare		Time working in the unit	
	Corr (r)	*p* value	Corr (r)	*p* value	Corr (r)	*p* value	Corr (r)	*p* value	Corr (r)	*p* value
**K1**	0.131	0.114	0.076	0.364	0.114	0.169	-0.003	0.975	-0.037	0.660
**K2**	-0.125	0.133	-0.034	0.683	-0.061	0.461	-0.168	0.043^*^	-0.050	0.547
**K3**	0.207	0.012^*^	0.196	0.018^*^	0.248	0.003^*^	0.129	0.121	-0.031	0.709
**K4**	0.080	0.335	0.080	0.341	0.143	0.085	0.113	0.175	0.116	0.163
**K5**	0.020	0.806	-0.067	0.423	-0.064	0.446	-0.100	0.230	-0.104	0.211
**K6**	0.094	0.258	0.189	0.023^*^	0.175	0.035^*^	0.063	0.447	-0.029	0.726
**K7**	0.050	0.546	0.146	0.079	0.122	0.142	0.096	0.247	0.027	0.744
**K8**	-0.049	0.559	0.074	0.375	0.040	0.635	0.076	0.361	0.029	0.725
**K9**	0.111	0.182	0.051	0.545	0.117	0.160	0.055	0.511	-0.038	0.649
**K10**	-0.179	0.031^*^	-0.117	0.160	-0.156	0.060	-0.168	0.042^*^	-0.188	0.023^*^
**K11**	0.110	0.188	0.046	0.581	0.025	0.769	0.010	0.909	0.072	0.389
**K12**	0.014	0.869	0.002	0.984	0.020	0.812	-0.049	0.556	0.087	0.298
**K13**	-0.104	0.214	-0.045	0.593	-0.096	0.250	-0.019	0.819	0.066	0.430
**K14**	-0.013	0.878	-0.075	0.367	-0.081	0.329	-0.012	0.884	-0.100	0.229
**K15**	-0.156	0.061	0.005	0.949	-0.035	0.679	0.011	0.899	-0.007	0.932
**K16**	0.181	0.029^*^	0.091	0.275	0.154	0.064	-0.005	0.956	0.047	0.576
**K17**	0.001	0.996	-0.060	0.474	-0.063	0.450	-0.118	0.158	-0.107	0.199
**K18**	0.108	0.194	-0.044	0.603	0.083	0.321	0.072	0.388	0.132	0.113
**K19**	0.123	0.139	0.106	0.203	0.121	0.146	0.060	0.469	-0.088	0.292
**K20**	0.031	0.712	-0.047	0.576	-0.074	0.375	-0.120	0.148	-0.029	0.731
**K21**	0.094	0.261	0.041	0.623	0.044	0.599	-0.006	0.939	-0.029	0.726
**K22**	-0.114	0.171	-0.081	0.331	-0.043	0.606	0.018	0.832	0.113	0.176
**K23**	-0.033	0.695	0.061	0.468	0.008	0.924	0.062	0.458	0.061	0.467
**SE1**	-0.107	0.198	-0.133	0.111	-0.072	0.387	-0.102	0.222	-0.044	0.595
**SE2**	0.073	0.378	-0.006	0.947	0.069	0.407	0.054	0.514	0.050	0.548
**SE3**	0.122	0.141	0.053	0.529	0.002	0.978	0.053	0.523	0.042	0.613
**SE4**	0.026	0.753	0.066	0.432	0.129	0.120	0.198	0.017^*^	0.081	0.334
**SE5**	0.031	0.710	-0.015	0.861	0.063	0.449	0.129	0.120	0.080	0.335
**SE6**	0.125	0.134	-0.028	0.735	0.033	0.691	0.016	0.846	-0.033	0.696
**SE7**	0.056	0.499	-0.044	0.599	0.022	0.795	-0.009	0.914	0.041	0.619
**SE8**	0.042	0.614	-0.035	0.680	0.079	0.346	0.031	0.712	0.046	0.584
**SE9**	0.086	0.304	-0.022	0.793	0.118	0.157	0.087	0.298	0.113	0.174
**SE10**	0.063	0.447	0.007	0.931	0.117	0.158	0.061	0.466	-0.018	0.832
**SE11**	0.160	0.053	0.129	0.121	0.181	0.029^*^	0.240	0.004^*^	0.101	0.226
**SE12**	0.085	0.309	-0.027	0.746	0.072	0.386	0.078	0.350	-0.003	0.973
**SE13**	0.113	0.175	-0.037	0.656	0.085	0.309	0.170	0.040^*^	0.175	0.035^*^
**SE14**	0.061	0.462	-0.014	0.864	0.143	0.084	0.099	0.233	0.025	0.768
**SE15**	0.020	0.808	0.029	0.729	0.108	0.196	0.009	0.916	0.092	0.270

Note: K = knowledge; SE = self-efficacy; Corr (r) = correlation;

* = P-value ≤ 0.05: _ = p-value close to 0.05.

In relation to academic training, 78.1% of participants had completed undergraduate courses at private institutions and 91.1% had completed graduate courses, the majority of which were at the specialization level (88.4%). Although 74% reported having previous contact with patients in PC, only 11.6% reported having specific training in the area (Table 1).

Table 2 presents the correlation analysis between the responses to BPW-BR and age, time since training, job tenure in health, job tenure in PHC and work time in the current work unit.

In the questions related to PC knowledge, positive correlations were observed between: item K2 and time working in PHC; item K3 and age, time since graduation and job tenure; item K6 and time since graduation and job tenure; item K10 and age, time working in PHC and job tenure in the unit; and item K16 and participants’ age. As for self-efficacy, positive correlations were observed between: SE4 and time working in PHC; SE11 and job tenure and working in PHC; and SE13 and time working in PHC and in the health unit (Table 2).

Table 3 presents the correlation data between each of BPW-BR responses and the categorical variables of gender, type of undergraduate institution, previous contact with patients in PC, and training in PC. Gender showed a significant positive correlation with items K1, K15, and K21, with no correlations observed with the self-efficacy items. The training institution had a positive correlation with items K10, K12, K17, and K20, in addition to items SE3, SE4, SE6, SE7, SE8, SE9, SE10, SE12, SE13, and SE14. Previous contact with patients in PC correlated positively with item K11 and items SE3, SE4, and SE5. No correlations were observed between training in PC and knowledge items; however, there was a positive correlation with items SE1 and SE4 (Table 3).

**Table 3:** Correlation of qualitative factors with the *Questionário de Conhecimento e Autoeficácia sobre Cuidados Paliativos* (BPW-BR) questions

Gen		M	SD	*p* value	Grad	M	SD	*p* value	Cont CP	M	SD	*p* value	F PC	M	SD	*p* value
K1	F	1.67	1.09	0.029^*^	PR	1.61	1.04	0.786	N	1.68	1.14	0.682	N	1.64	1.00	0.150
	M	1.21	0.72		PB	1.56	1.08		Y	1.56	1.02		Y	1.29	1.00	
K2	F	1.85	1.11	0.699	PR	1.84	1.08	0.557	N	1.82	1.09	0.907	N	1.81	1.00	0.525
	M	1.67	0.87		PB	1.75	1.08		Y	1.82	1.08		Y	1.94	2.00	
K3	F	2.62	1.24	0.570	PR	2.72	1.24	0.193	N	2.76	1.26	0.484	N	2.71	3.00	0.109
	M	2.79	1.25		PB	2.41	1.24		Y	2.61	1.24		Y	2.18	1.00	
K4	F	2.92	1.17	0.437	PR	2.87	1.19	0.128	N	2.97	1.20	0.829	N	2.92	3.00	0.718
	M	3.00	1.35		PB	3.16	1.22		Y	2.92	1.20		Y	3.00	4.00	
K5	F	3.94	0.23	0.569	PR	3.91	0.34	0.414	N	3.87	0.41	0.193	N	3.93	4.00	0.323
	M	3.83	0.56		PB	3.97	0.18		Y	3.94	0.27		Y	3.88	4.00	
K6	F	3.39	0.87	0.891	PR	3.44	0.81	0.357	N	3.50	0.69	0.640	N	3.42	4.00	0.405
	M	3.42	0.78		PB	3.25	0.98		Y	3.36	0.90		Y	3.24	3.00	
K7	F	2.30	1.19	0.073	PR	2.39	1.22	0.791	N	2.26	1.18	0.464	N	2.40	3.00	0.762
	M	2.79	1.18		PB	2.34	1.12		Y	2.43	1.21		Y	2.29	2.00	
K8	F	3.94	0.23	0.630	PR	3.96	0.21	0.093	N	3.92	0.27	0.607	N	3.95	4.00	0.309
	M	3.92	0.28		PB	3.88	0.34		Y	3.94	0.23		Y	3.88	4.00	
K9	F	1.79	0.95	0.963	PR	1.76	0.94	0.572	N	1.97	1.00	0.138	N	1.77	1.00	0.627
	M	1.75	0.90		PB	1.84	0.92		Y	1.71	0.91		Y	1.88	2.00	
K10	F	2.51	1.31	0.347	PR	2.57	1.32	0.038^*^	N	2.71	1.29	0.179	N	2.49	3.00	0.432
	M	2.21	1.35		PB	2.06	1.22		Y	2.37	1.32		Y	2.24	2.00	
K11	F	1.23	0.67	0.768	PR	1.25	0.69	0.698	N	1.42	0.89	0.049^*^	N	1.25	1.00	0.275
	M	1.21	0.51		PB	1.16	0.45		Y	1.16	0.51		Y	1.06	1.00	
K12	F	3.07	0.92	0.411	PR	3.17	0.93	0.016^*^	N	3.21	0.87	0.353	N	3.06	3.00	0.389
	M	3.21	0.93		PB	2.81	0.86		Y	3.05	0.94		Y	3.29	3.00	
K13	F	2.62	1.26	0.360	PR	2.63	1.24	0.376	N	2.79	1.26	0.481	N	2.67	3.00	0.916
	M	2.92	1.10		PB	2.81	1.26		Y	2.63	1.23		Y	2.65	3.00	
K14	F	2.01	0.97	0.640	PR	2.04	1.00	0.896	N	2.03	0.97	0.975	N	2.00	2.00	0.308
	M	2.13	1.08		PB	2.00	0.95		Y	2.03	1.00		Y	2.24	2.00	
K15	F	3.95	0.31	0.009^*^	PR	3.91	0.37	0.125	N	3.89	0.51	0.973	N	3.94	4.00	0.233
	M	3.83	0.38		PB	4.00	0.00		Y	3.94	0.23		Y	3.88	4.00	
K16	F	2.61	1.15	0.407	PR	2.68	1.17	0.343	N	2.89	1.01	0.147	N	2.68	3.00	0.187
	M	2.83	1.09		PB	2.50	1.05		Y	2.56	1.18		Y	2.35	3.00	
K17	F	2.10	1.12	0.561	PR	2.22	1.17	0.004^*^	N	2.16	1.13	0.574	N	2.12	2.00	0.142
	M	1.96	1.20		PB	1.56	0.84		Y	2.05	1.14		Y	1.71	1.00	
K18	F	1.55	0.95	0.357	PR	1.66	1.02	0.088	N	1.79	1.07	0.082	N	1.61	1.00	0.256
	M	1.75	1.03		PB	1.31	0.69		Y	1.51	0.92		Y	1.35	1.00	
K19	F	1.31	0.72	0.440	PR	1.39	0.86	0.573	N	1.29	0.77	0.427	N	1.36	1.00	0.366
	M	1.54	1.06		PB	1.19	0.40		Y	1.37	0.79		Y	1.24	1.00	
K20	F	1.60	0.96	0.179	PR	1.78	1.10	0.019^*^	N	1.71	1.01	0.484	N	1.70	1.00	0.337
	M	2.04	1.33		PB	1.28	0.68		Y	1.66	1.05		Y	1.47	1.00	
K21	F	1.61	1.02	0.019^*^	PR	1.79	1.12	0.117	N	1.82	1.14	0.396	N	1.71	1.00	0.923
	M	2.21	1.25		PB	1.44	0.88		Y	1.68	1.06		Y	1.76	1.00	
K22	F	3.07	1.16	0.415	PR	3.00	1.17	0.319	N	2.89	1.23	0.357	N	2.98	3.00	0.116
	M	2.83	1.31		PB	3.16	1.22		Y	3.08	1.17		Y	3.41	4.00	
K23	F	3.32	1.04	0.861	PR	3.29	1.05	0.346	N	3.34	1.02	0.975	N	3.32	4.00	0.944
	M	3.42	0.93		PB	3.50	0.92		Y	3.33	1.02		Y	3.47	4.00	
SE1	F	3.61	0.80	0.474	PR	3.61	0.78	0.523	N	3.61	0.68	0.565	N	3.54	4.00	0.009^*^
	M	3.54	0.78		PB	3.53	0.84		Y	3.59	0.83		Y	4.00	4.00	
SE2	F	3.49	0.89	0.617	PR	3.51	0.91	0.441	N	3.45	1.06	0.774	N	3.46	4.00	0.083
	M	3.54	0.93		PB	3.47	0.84		Y	3.52	0.84		Y	3.82	4.00	
SE3	F	3.63	0.72	0.923	PR	3.72	0.68	<0.001^*^	N	3.32	1.02	0.024^*^	N	3.60	4.00	0.201
	M	3.54	0.93		PB	3.25	0.88		Y	3.72	0.61		Y	3.76	4.00	
SE4	F	3.41	0.94	0.485	PR	3.29	1.02	0.020^*^	N	2.97	1.20	0.007^*^	N	3.30	4.00	0.005^*^
	M	3.21	1.14		PB	3.69	0.74		Y	3.52	0.85		Y	3.94	4.00	
SE5	F	3.63	0.68	0.580	PR	3.66	0.64	0.334	N	3.34	0.94	0.018^*^	N	3.64	4.00	0.469
	M	3.54	0.78		PB	3.47	0.88		Y	3.71	0.56		Y	3.47	4.00	
SE6	F	3.43	0.84	0.092	PR	3.55	0.77	0.033^*^	N	3.32	0.99	0.284	N	3.44	4.00	0.265
	M	3.63	0.88		PB	3.16	1.05		Y	3.52	0.79		Y	3.65	4.00	
SE7	F	3.74	0.56	0.623	PR	3.82	0.50	<0.001^*^	N	3.55	0.80	0.080	N	3.73	4.00	0.318
	M	3.79	0.51		PB	3.47	0.62		Y	3.81	0.41		Y	3.88	4.00	
SE8	F	3.31	0.85	0.249	PR	3.39	0.87	<0.001^*^	N	3.16	0.95	0.391	N	3.22	3.00	0.064
	M	3.04	1.04		PB	2.84	0.85		Y	3.31	0.87		Y	3.59	4.00	
SE9	F	3.26	0.99	0.524	PR	3.33	1.01	0.003^*^	N	3.05	1.11	0.215	N	3.23	4.00	0.718
	M	3.08	1.18		PB	2.88	0.98		Y	3.30	0.98		Y	3.24	4.00	
SE10	F	3.11	1.03	0.117	PR	3.32	0.93	0.001^*^	N	2.97	1.22	0.422	N	3.13	3.00	0.174
	M	3.46	0.88		PB	2.66	1.12		Y	3.24	0.93		Y	3.47	4.00	
SE11	F	3.34	1.06	0.348	PR	3.46	1.00	0.056	N	3.50	0.86	0.688	N	3.40	4.00	0.434
	M	3.58	0.83		PB	3.13	1.10		Y	3.34	1.08		Y	3.24	4.00	
SE12	F	3.43	0.92	0.582	PR	3.53	0.87	0.017^*^	N	3.39	0.97	0.830	N	3.43	4.00	0.374
	M	3.54	0.83		PB	3.19	0.97		Y	3.47	0.88		Y	3.59	4.00	
SE13	F	3.49	0.78	0.519	PR	3.61	0.72	0.001^*^	N	3.32	0.96	0.176	N	3.49	4.00	0.753
	M	3.58	0.78		PB	3.16	0.88		Y	3.57	0.70		Y	3.65	4.00	
SE14	F	3.52	0.77	0.395	PR	3.55	0.80	0.011^*^	N	3.24	1.00	0.077	N	3.47	4.00	0.863
	M	3.29	1.04		PB	3.22	0.87		Y	3.56	0.74		Y	3.53	4.00	
SE15	F	3.94	0.23	0.630	PR	3.96	0.21	0.093	N	3.92	0.27	0.607	N	3.93	4.00	0.263
	M	3.92	0.28		PB	3.88	0.34		Y	3.94	0.23		Y	4.00	4.00	

Note: C = knowledge; SE = self-efficacy; M = mean; SD = Standard Deviation; Grad = undergraduate institution; Cont PC = contact with patients in palliative care; F = female; M = male; PR = private; PB = public; N = no; Y = yes; PC = training in palliative care;

* = p-value ≤ 0.05; _ = p-value close to 0.05. Source: own elaboration.

**Table 4:** Comparison between the answers given by participants according to their profession

	Profession	Mean	Standard Deviation	*p* value		Profession	Mean	Standard Deviation	*p* value
**KNOWLEDGE**									
**K1**	Nurse	1.75	1.18	0.183	**K2**	Nurse	1.98	1.12	0.013^*^
	Physician	1.29	0.76			Physician	1.29	0.60	
	Others	1.57	0.98			Others	1.91	1.14	
**K3**	Nurse	2.78	1.29	0.319	**K4**	Nurse	2.91	1.16	0.012^*^
	Physician	2.46	1.10			Physician	3.46	0.96	
	Others	2.59	1.25			Others	2.69	1.27	
**K5**	Nurse	3.97	0.18	0.371	**K6**	Nurse	3.53	0.71	0.046^*^
	Physician	3.89	0.42			Physician	2.96	1.14	
	Others	3.89	0.37			Others	3.46	0.77	
**K7**	Nurse	2.42	1.18	0.023^*^	**K8**	Nurse	3.98	0.13	0.122
	Physician	1.86	1.04			Physician	3.89	0.31	
	Others	2.61	1.23			Others	3.91	0.29	
**K9**	Nurse	1.75	0.94	0.720	**K10**	Nurse	2.55	1.31	0.809
	Physician	1.68	0.82			Physician	2.39	1.34	
	Others	1.87	0.99			Others	2.39	1.32	
**K11**	Nurse	1.25	0.64	0.464	**K12**	Nurse	3.08	0.93	0.464
	Physician	1.11	0.42			Physician	2.93	0.98	
	Others	1.26	0.73			Others	3.19	0.89	
**K13**	Nurse	2.41	1.29	0.075	**K14**	Nurse	1.95	0.93	0.537
	Physician	2.86	1.15			Physician	1.96	1.07	
	Others	2.89	1.18			Others	2.15	1.02	
**K15**	Nurse	3.88	0.45	0.147	**K16**	Nurse	2.95	1.09	0.002^*^
	Physician	4.00	0.00			Physician	2.07	1.09	
	Others	3.96	0.19			Others	2.57	1.13	
**K17**	Nurse	2.19	1.13	0.032^*^	**K18**	Nurse	1.70	1.05	0.073
	Physician	1.57	0.92			Physician	1.18	0.48	
	Others	2.20	1.19			Others	1.65	1.01	
**K19**	Nurse	1.41	0.90	0.990	**K20**	Nurse	1.89	1.13	0.005^*^
	Physician	1.25	0.52			Physician	1.18	0.61	
	Others	1.33	0.75			Others	1.67	1.03	
**K21**	Nurse	1.78	1.12	0.682	**K22**	Nurse	2.97	1.15	0.059
	Physician	1.61	1.07			Physician	3.43	1.10	
	Others	1.69	1.04			Others	2.91	1.23	
**K23**	Nurse	3.30	1.05	0.090					
	Physician	3.71	0.60						
	Others	3.19	1.12						
**SELF-KNOWLEDGE**									
**SE1**	Nurse	3.72	0.63	0.032^*^	**SE2**	Nurse	3.69	0.66	0.004^*^
	Physician	3.75	0.65			Physician	3.79	0.50	
	Others	3.37	0.98			Others	3.13	1.15	
**SE3**	Nurse	3.73	0.60	0.297	**SE4**	Nurse	3.47	0.85	<0.001^*^
	Physician	3.39	0.99			Physician	3.89	0.31	
	Others	3.59	0.77			Others	3.00	1.18	
**SE5**	Nurse	3.61	0.70	0.596	**SE6**	Nurse	3.52	0.80	0.393
	Physician	3.71	0.66			Physician	3.29	0.94	
	Others	3.57	0.72			Others	3.50	0.86	
**SE7**	Nurse	3.80	0.57	0.266	**SE8**	Nurse	3.48	0.76	0.024^*^
	Physician	3.71	0.46			Physician	2.96	1.04	
	Others	3.70	0.57			Others	3.17	0.91	
**SE9**	Nurse	3.53	0.78	0.009^*^	**SE10**	Nurse	3.25	0.94	0.320
	Physician	2.96	0.96			Physician	3.00	0.94	
	Others	3.02	1.21			Others	3.17	1.13	
**SE11**	Nurse	3.78	0.58	<0.001^*^	**SE12**	Nurse	3.70	0.68	0.004^*^
	Physician	3.14	1.11			Physician	3.50	0.69	
	Others	3.04	1.23			Others	3.13	1.12	
**SE13**	Nurse	3.63	0.72	0.179	**SE14**	Nurse	3.58	0.75	0.375
	Physician	3.43	0.79			Physician	3.43	0.74	
	Others	3.41	0.84			Others	3.39	0.94	
**SE15**	Nurse	3.95	0.21	0.530					
	Physician	3.89	0.31						
	Others	3.94	0.23						

Note: K = knowledge; SE = self-efficacy;

* = p-value ≤ 0.05; _= p-value close to 0.05.

To assess possible differences in responses to BPW-BR between professional categories, a comparative analysis was performed, as described in Table 4. Participants were grouped into three categories, such as “nursing”, “medicine” and “other professionals”, in order to ensure sufficient proportionality for statistical analysis. Significant differences were observed between groups in questions K2, K4, K6, K7, K16, K17 and K20, related to knowledge, and SE1, SE2, SE4, SE8, SE9, SE11 and SE12, related to self-efficacy (Table 4).

## DISCUSSION

Understanding what the multidisciplinary PHC team knows about PC, as well as their beliefs about their ability to care for this type of patient, is of utmost importance for planning training on the topic and for developing and improving public policies, such as PNCP. Such understanding can contribute to providing earlier and higher-quality care to people in need of PC in entry-level and health promotion services, as proposed by PHC^(4)^.

PNCP approval represents a significant advance, since it will allow the allocation of specific financial and educational resources for actions that promote PC in PHC^(7,18)^, reinforcing the relevance of studies such as this one, which support educational strategies by identifying practical and learning needs of professionals, enabling the direction and optimization of resources.

Most participants in this study (74%) reported having contact with patients in PC, although 88.4% did not have specific training on the subject (Table 1). Although literature on the inclusion of PC in PHC is still scarce, there is a consensus that the lack of formal knowledge about PC can hinder the early identification of eligible cases, especially in contexts where there is no malignancy involved or among younger patients^(19)^. With the implementation of PNCP, it is expected that new training courses on PC aimed at PHC professionals will be created, in addition to the adoption of algorithms, instruments and strategies for the early identification of eligible patients as well as greater clarity about the care flows in RAS^(7,18)^.

Among the findings of this study, it was observed that older professionals with more time since graduation and experience demonstrated more knowledge about medication administration, the importance of family presence at the end of life, and the PC philosophy. Those with more time working in PHC showed better performance in self-efficacy for communicating with physicians about the need for PC, performing adequate oral hygiene at the end of life, and recognizing mental health problems in patients (Table 2). These results support the literature, which indicates that more experienced professionals tend to feel more secure and confident in their duties when compared to younger professionals^(20)^. Other studies also demonstrate that longer time working in PHC is directly related to greater knowledge about PC^(9,21)^. Having more experienced people in training can be an interesting experience, in addition to providing an intergenerational exchange of knowledge.

Primary care professionals who reported previous contact with patients undergoing PC showed a better understanding of the influence of emotions on pain intensity and greater self-efficacy in communicating with patients, family members, and the medical team (Table 3). Effective communication among all those involved in PC favors more assertive behaviors, providing comfort and enabling the development of effective action plans. Although challenging, especially for physicians, communication is an essential skill that can and should be developed, constituting a key point in training aimed at working in PC in PHC^(22)^.

Unlike international studies^(9,21,22)^, no correlation was observed between knowledge about PC and the fact that professionals had received previous training in the area. However, a correlation was identified between self-efficacy and data collection on pain and communication with physicians about PC prescription. Although the literature extensively addresses PC knowledge, little is discussed about the role of self-efficacy in good care practices. It is known that greater knowledge promotes greater self-efficacy, and this, in turn, can contribute to the provision of better quality care^(11,23,24)^. Therefore, it is recommended that training include, in addition to theoretical content, practical and planning actions^(12)^, through in-person internships and discussions of clinical cases, so that professionals feel more confident in clinical practice.

The analysis of participants’ responses according to the type of training institution revealed that those trained in private institutions (78%) had better knowledge about the PC philosophy and communication of prognosis to patients. They also obtained higher scores on questions related to self-efficacy in communication with patients and family members, care management, and provision of complementary therapies. Professionals trained in public institutions demonstrated greater knowledge about teamwork in PC and opioid administration, in addition to feeling more prepared to talk to the family physician about the need for PC (Table 3).

The inclusion of PC in the training of healthcare professionals is still recent. In Brazil, as of 2022, it became mandatory to offer a specific course on PC in medical courses^(25)^. A study with 2,225 recently graduated Brazilian physicians found that 99.2% consider the inclusion of PC in undergraduate courses to be important; however, only 46.2% reported having received training on the topic, with those from private institutions reporting greater exposure to PC training than those from public institutions (57% versus 51%)^(26)^. These data highlight the relevance of PC training during undergraduate courses. In other health courses, this training is not yet mandatory, but it constitutes an essential step that could change the perception that other professionals have about their role in PC, in addition to adding a lot to patient care, who essentially need multidisciplinary care.

When comparing professional categories, physicians showed greater knowledge and self-efficacy regarding pharmacological pain management and pain data collection, including opioid prescription, in line with studies conducted in Spain, Vietnam, and Malaysia^(9,10,27)^. However, such studies indicate that this knowledge is more evident among professionals with previous experience in hospitals or specialized PC services, in addition to evidencing the restriction on access to medications such as morphine, a reality similar to that in Brazil^(28)^. It is important to highlight that, in Brazil, only physicians are authorized to prescribe opioids, which may justify the greater knowledge among these professionals^(28)^.

Physicians also reported greater self-efficacy in providing guidance on nausea management and convincing family physicians of the need for PC. However, as already identified in literature, they demonstrated lower performance in identifying complex patient needs and in valuing family presence in the finitude process (Table 4). An Australian systematic review indicated that, although they recognize the importance of communication at the end of life, physicians feel unprepared and poorly trained, encountering structural difficulties in this regard, including communication failures among team members, which negatively impacts interactions with patients and family members^(29)^. Given that communication is not an innate skill, it is important to teach, practice, and constantly improve it, aiming at qualifying PC care.

Difficulties in offering complementary relaxation therapies and limited knowledge about constipation as a secondary symptom were demonstrated among physicians (Table 4). In a systematic review that included data from Brazil, it was observed that, although PHC physicians are the most open to integrative and complementary practices, their acceptance is around 65%^(30)^. Although there is positive evidence about practices such as acupuncture, reflexology and massage in PC^(31)^, many physicians show little interest in specializing in them, delegating their application to other team members.

Nurses, in general, presented the best indicators of self-efficacy in PC conduct, especially in identifying patients’ needs, offering complementary therapies, oral hygiene and guidance on medication side effects. They also demonstrated better performance in communicating with patients and family members, although they had less knowledge about the use of transdermal patches and prescription of opioids (Table 4). The literature indicates that, although nurses’ theoretical knowledge about PC principles still needs to be strengthened, their conduct and attitudes in caring for terminally ill patients are a differential^(9,13,21)^, which supports the findings of this study.

The role of nurses in healthcare, especially in PC, is strongly related to case management, communication with family members, ongoing support, and pain management through pharmacological and non-pharmacological strategies^(32)^. Therefore, it is essential that training expands the theoretical foundations of PC, including addressing its concepts and principles, points to be improved in practice, already identified in several national and international studies^(9,11-13,21,23)^, as well as in this work. Nursing can play an important role in matrixing the rest of the team in PHC, since it has more experience in these key points for PC.

The other healthcare professionals, who were not physicians or nurses, performed significantly worse on several knowledge questions and, above all, self-efficacy questions, highlighting the self-reported difficulty in suggesting PC prescriptions to physicians. Together with nurses, they also performed worse on question K17, which addresses the prioritization of physician aspects over other interventions, indicating that multidisciplinary team professionals are unable to recognize and value their contributions to the team (Table 4).

The American Society of Clinical Oncology recognizes that PC should be provided by interprofessional teams, citing professionals such as social workers, physical therapists, speech therapists, nutritionists, psychologists, and occupational therapists, all of whom were included in this study^(31)^. However, the guideline is limited to brief mentions, without presenting specific evidence on each professional area, with its recommendations being more focused on the physical and psychological dimensions. Studies with psychologists in PC are more frequent and indicate that their work should encompass both the patient and their family and support the interprofessional team^(33)^. Although there are also studies on physical therapists^(34)^ and social workers^(35)^ in PC, there is no specific research on their work in PHC, which reinforces the need to investigate these categories in greater depth in order to support the development of appropriate instruments and training. Therefore, this study chose BPW-BR, which was validated in the Brazilian context and with several professionals^(17)^.

Conceptually, PC requires a team approach^(36)^. However, in PHC, there are difficulties in effective team integration, communication between professionals and exchange of knowledge. Furthermore, the very configuration of PHC in Brazil results in a lower presence of these professionals, limiting their participation in studies^(4,8)^. In the present study, we had difficulty in deepening the analyses in each category separately, precisely because there is a reduced number of these professionals in PHC teams.

Although interprofessionality promotion is a principle of PHC, it is still observed that performance is predominantly unilateral^(37)^. The lack of knowledge about PC by professionals also compromises the recognition of the demands of patients and their families, making it difficult to indicate conduct and technical arguments about PC with the team^(38)^. Therefore, studies with more representative samples of these categories are needed to verify whether BPW-BR, in its current format, is adequate to assess the interprofessional PHC team’s knowledge and self-efficacy in PC and to verify, more specifically, which points need to be improved in each professional category.

The findings of this study support evidence in literature regarding the points to be improved in PC training for all healthcare professionals working in PHC. Specific training in this area is strongly recommended, both to promote theoretical and philosophical mastery of PC and to develop skills in communication, pain management (pharmacological and non-pharmacological) and promotion of interprofessionality in care.

### Study limitations

As limiting factors of this research, we can consider the small number of participants, which was especially due to the high demand of healthcare professionals involved in treating COVID-19 cases or even those infected by the virus during the collection period^(39)^. In addition, there is low regional variability in professionals’ work, since they all work in the city of São Paulo, making it impossible to extrapolate these results to other regions of Brazil without other studies being carried out.

The reduced number of professionals included in the “Others” category made it impossible to analyze data with greater distinction between each of the professions, but we strongly recommend that more research be carried out with these professional categories, including to understand whether BPW-BR is a good instrument for analyzing their knowledge and self-efficacy about PC.

It is important to be clear about the internal consistency of the “knowledge” construct in BPW-BR validity, whose Cronbach’s alpha was 0.486^(17)^, a value considered median^(40)^, suggesting the importance of validating BPW-BR with a larger number of participants, from different regions of Brazil, to verify whether there are structural problems of internal consistency in items on knowledge or whether it was a value biased by the sample number.

### Contributions to health

As contributions to the health field, it was possible to obtain a better understanding of what PHC professionals know and feel skilled and capable of performing in PC. Through this information, it is possible to design more objectively training programs specific to PHC professionals’ demands. The study was pioneering in providing information on other healthcare professionals other than physicians and nurses, making it clear that it is necessary to expand research with other professional categories that are fundamental in the provision of PC and need to be an active part of decisions and interventions.

As possible strategies to educate professionals about PC in PHC, we suggest that the subject be discussed in matrix meetings and continuing education programs, and that the implementation of PNCP be used to request training, specializations and funds to improve infrastructure and hire staff. More education on the subject will also help identify patients eligible for PC, improving quality of life and providing more information to the population served.

## CONCLUSIONS

Based on the sociodemographic data and the results of BPW-BR, it was possible to outline an overview of knowledge and self-efficacy in PC among the different professionals who make up the PHC teams. In general, all categories presented unsatisfactory knowledge and self-efficacy on the topic.

The points to be improved in clinical practice were communication skills between teams and with patients and family members, pain management, technical and conceptual knowledge of PC and understanding of the role of each healthcare professional within PC in PHC.

## Data Availability

Available research data is in the body of the article.
