# Supplementary material for: Primary healthcare professionals’ knowledge and self-efficacy on palliative care
Source: Rev Bras Enferm. 2025 Nov 28;78(5):e20240056. doi: 10.1590/0034-7167-2024-0056 (PMC12673609; doi:10.1590/0034-7167-2024-0056)
Supplement: Translation

# Translation: Conhecimento e autoeficácia de profissionais da Atenção Primária à Saúde sobre cuidados paliativos

**DOI:** 10.1590/0034-7167-2024-0056pt

**Authors:** Elisa Cavalheiro Libardi, Beatriz Aparecida Ozello Gutierrez

**Affiliations:** I Universidade de São Paulo. São Paulo, SP, Brasil

**Keywords:** Cuidados Paliativos, Conhecimento, Autoeficácia, Atenção Primária à, Saúde, Equipe Multiprofissional.

## Abstract

**Objetivo:**

obter informações referentes a conhecimento e autoeficácia sobre cuidados paliativos de profissionais que trabalham na Atenção Primária à Saúde.

**Métodos:**

foram avaliados 146 profissionais de diversas profissões por meio de questionário sociodemográfico e profissional e por meio do Questionário de Conhecimento e Autoeficácia Sobre Cuidados Paliativos (BPW-BR).

**Resultados:**

há déficits importantes no conhecimento e autoeficácia por parte de todos os profissionais da Atenção Primária à Saúde sobre cuidados paliativos. Conhecimento teórico e prático, habilidades de comunicação, manejo farmacológico e não farmacológico de dor e melhor integração de conhecimentos entre equipe de Atenção Primária à Saúde sobre cuidados paliativos são necessários.

**Conclusões:**

o estudo mostrou o panorama de como estão o conhecimento e a autoeficácia sobre cuidados paliativos de profissionais que compõem as equipes da Atenção Primária à Saúde, mas precisa ser ampliado em número, variedade de participantes e alcance regional, para resultados que expressem a realidade brasileira.

## INTRODUÇÃO

De acordo com a organização dos níveis de atenção à saúde no Sistema Único de Saúde (SUS), a Atenção Primária à Saúde (APS) é responsável por coordenar e ordenar o cuidado, sendo a principal porta de entrada dos usuários no sistema de saúde. Cabe à APS resolver a maioria dos problemas de saúde, encaminhando os usuários aos demais níveis de atenção apenas em situações de maior complexidade ou que demandem recursos tecnológicos não disponíveis nesse nível assistencial^(1)^.

Nos últimos anos, os cuidados paliativos (CP) passaram a integrar os serviços ofertados pela APS^(2)^. Os CP consistem em um conjunto de intervenções destinadas a indivíduos de qualquer faixa etária e seus familiares acometidos por doenças que ameaçam a continuidade da vida. Seu principal objetivo é o alívio do sofrimento, sendo indicados a pessoas com diagnóstico de doenças crônicas progressivas e prognóstico desfavorável, que geram sofrimento físico, emocional, social e espiritual tanto para os pacientes quanto para seus cuidadores e rede de apoio^(3)^.

A inserção dos CP na APS é uma estratégia promissora para a identificação precoce e o acompanhamento longitudinal dos pacientes elegíveis desde o momento do diagnóstico. Além disso, favorece a aproximação geográfica, emocional e cultural entre as equipes de saúde, os pacientes e suas famílias, facilitando a manutenção do cuidado no domicílio^(4)^.

Apesar do aumento da demanda por CP, o Brasil ainda apresenta desempenho insatisfatório no cenário internacional, com número insuficiente de serviços especializados^(5)^ e controle da dor abaixo do ideal, comprometendo a qualidade de vida e de morte^(6)^. Entretanto, iniciativas de fortalecimento vêm sendo implementadas, destacando-se, em maio de 2024, a instituição da Política Nacional de Cuidados Paliativos (PNCP). Essa política visa ampliar o financiamento em todas as esferas de gestão, capacitar profissionais de saúde, expandir equipes interdisciplinares especializadas, fomentar a educação da população sobre CP e garantir sua implementação em toda a Rede de Atenção à Saúde (RAS) ^(7)^.

A qualidade da assistência em CP está diretamente relacionada à atuação de equipes interprofissionais e interdisciplinares, capacitadas técnica e cientificamente, com habilidades clínicas que contemplem as múltiplas dimensões do sofrimento - físico, emocional, espiritual e social - dos pacientes e de suas redes de apoio^(8)^. A APS, por sua característica organizacional, é um ambiente propício à atuação dessas equipes, desde que os profissionais possuam conhecimento adequado sobre a temática^(8)^.

Estudos internacionais demonstram níveis insatisfatórios de conhecimento e autoeficácia em CP entre profissionais da APS, tanto em países desenvolvidos quanto em desenvolvimento. Na Espanha, por exemplo, avaliação realizada com 561 médicos e enfermeiros da APS revelou que 65,5% dos enfermeiros e 32,5% dos médicos apresentaram conhecimento fraco ou insuficiente. No entanto, profissionais que haviam participado de formações contínuas, cursos de especialização ou que tiveram contato com CP ainda na graduação apresentaram desempenho significativamente superior^(9)^.

Na Malásia, país onde os CP também têm implantação recente na APS, estudo com 271 médicos identificou que apenas 12,6% haviam recebido alguma formação, mesmo que pontual, sobre o tema. Os resultados demonstraram correlação positiva entre maior conhecimento e condutas mais adequadas em CP^(10)^, reforçando a importância de investigações que identifiquem dificuldades e potencialidades no conhecimento dos profissionais da APS, com vistas à construção de estratégias formativas mais eficazes.

A autoeficácia em CP também é um aspecto fundamental, pois influencia diretamente a qualidade da assistência prestada^(11)^. Embora ainda pouco explorada, essa dimensão revela dados relevantes. Estudo realizado no sul dos Estados Unidos da América evidenciou que enfermeiros com maior autoeficácia em CP se sentem mais preparados para discutir o processo de morrer, oferecer suporte ao paciente e familiares, e manejar sintomas como delirium, dor e alterações respiratórias^(11)^. A baixa autoeficácia, por sua vez, compromete o planejamento das ações e a identificação de pacientes elegíveis para CP^(12)^.

No Brasil, ainda há escassez de estudos que investiguem, de forma sistemática, o conhecimento e a autoeficácia de profissionais da APS em relação aos CP - uma lacuna preocupante, devido ao crescimento da área e à necessidade de qualificação contínua. Pesquisa realizada em Minas Gerais revelou que enfermeiros da APS apresentam conhecimento limitado, especialmente no que se refere à definição e aos princípios filosóficos dos CP, apontando a urgência de investimentos em formação profissional^(13)^.

Além da quantidade reduzida de estudos, observa-se também uma restrita diversidade nas categorias profissionais investigadas. As pesquisas se concentram, majoritariamente, em médicos e enfermeiros, sendo raros os estudos que incluam assistentes sociais e inexistentes aqueles que abranjam os demais integrantes das equipes multiprofissionais^(14)^.

Diante desse cenário, destaca-se a necessidade de ampliar as investigações sobre conhecimento e autoeficácia em CP entre os profissionais da APS, considerando a diversidade de formações. Isso permitirá a formulação de estratégias educativas mais assertivas, favorecendo a qualificação da atenção paliativa nesse nível do cuidado em saúde.

## OBJETIVOS

Objetivo geral: avaliar o nível de conhecimento e autoeficácia em CP da equipe multiprofissional que compõem a rede de APS no município de São Paulo.

Objetivo específico: identificar, por meio das respostas do Questionário de Conhecimento e Autoeficácia sobre Cuidados Paliativos (BPW-BR), os pontos a serem melhorados na prática dos profissionais de saúde com ensino superior atuantes nas unidades de APS do município de São Paulo, considerando os domínios de dor, controle de sintomas, conhecimento geral sobre o tema e atitudes sobre a morte.

## MÉTODOS

### Aspectos éticos

O estudo foi conduzido de acordo com as diretrizes de ética nacionais e internacionais, identificado pelo Certificado de Apresentação para Apreciação Ética n^o^ 52538721.3.3001.0086, sendo aprovado pelos Comitês de Ética em Pesquisa da Secretaria Municipal de Saúde do Município de São Paulo e da Escola de Artes, Ciências e Humanidades da Universidade de São Paulo, sob Parecer n^o^ 5.096.355, que está anexado à presente submissão.

Todos os participantes foram previamente informados sobre os procedimentos a serem realizados e instruídos a preencher o Termo de Consentimento Livre e Esclarecido (TCLE), que viabilizou a obtenção de dados para pesquisa e publicação. Os profissionais receberam uma via do documento por e-mail, automaticamente, após o preenchimento do TCLE.

### Desenho, período e local do estudo

Trata-se de estudo observacional transversal exploratório com análise quantitativa de dados, com avaliação de profissionais de saúde que trabalhavam em unidades vinculadas à APS das regiões de saúde sudeste, leste e norte do município de São Paulo, SP, Brasil, em Unidades Básicas de Saúde (UBS) e Unidades Básicas de Saúde com Assistência Médica Ambulatorial integradas (UBS/AMA integradas). O período de coleta de dados foi entre fevereiro de 2022 e dezembro de 2022.

Para a construção do estudo, foi utilizado o *Consensus Reporting Items for Studies in Primary Care-CRISP Statement*, que tem como objetivo nortear pesquisas realizadas na APS^(15)^.

### Amostra e critérios de inclusão e exclusão

A amostra foi composta por 146 profissionais de saúde com formação em nível superior, vinculados à APS, atuantes nos programas Estratégia Saúde da Família (ESF), Núcleo de Apoio à Saúde da Família (NASF), Equipe Multiprofissional de Atenção Domiciliar (EMAD) e Equipe Multidisciplinar de Apoio (EMAP). Foram incluídos apenas os profissionais que consentiram em participar da pesquisa. Foram excluídos aqueles que, no momento da coleta de dados, não dispunham de tempo hábil para responder ao instrumento, estavam afastados por licença médica ou férias, ou que consultaram outras pessoas ou materiais informativos durante o preenchimento do questionário.

### Protocolo do estudo

A coleta de dados foi realizada por meio de formulário eletrônico (*Google Forms^®^*), contendo dois instrumentos: um questionário de caracterização sociodemográfica e profissional e o BPW-BR.

O questionário sociodemográfico contemplou informações sobre idade, sexo, religião e estado civil. As variáveis profissionais incluíram tipo e tempo de formação, realização de pós-graduação, tempo de atuação na unidade, experiência prévia com CP e participação em cursos ou treinamentos sobre o tema.

O BPW-BR é composto por 38 itens, sendo 23 destinados à avaliação do conhecimento dos profissionais sobre CP, abordando tópicos como manejo da dor, controle de sintomas, conhecimento geral e atitudes diante da morte, e 15 itens voltados à análise da autoeficácia na prestação dos CP. Os itens são apresentados em escala de Likert de quatro pontos: 4 - “verdadeiro”; 3 - “mais ou menos verdadeiro”; 2 - “dificilmente verdadeiro”; e 1 - “não é verdadeiro”^(16)^. Desenvolvido originalmente na Alemanha^(16)^, o BPW-BR foi adaptado e validado para o contexto da APS no Brasil com profissionais de diferentes áreas da saúde^(17)^ (Quadro 1).

**Quadro 1:** Perguntas do Questionário de Conhecimento e Autoeficácia sobre Cuidados Paliativos (BPW-BR)

Questionário de Conhecimento e Autoeficácia sobre Cuidados Paliativos (BPW-BR)	
Escala de Likert: Verdadeiro=4; Mais ou menos verdadeiro=3; Dificilmente verdadeiro=2; Não é verdadeiro=1	
**C1-** Cuidados Paliativos nunca devem ser combinados com tratamentos curativos.	**RE=1**
**C2 -** Medicamentos anti-inflamatórios não esteróides devem ser excluídos durante o uso esporádico de opióides.	**RE=1**
**C3 -** Administração de fluidos por via subcutânea (hipodermóclise) é necessária para aliviar a boca seca (xerostomia) em pacientes em final de vida.	**RE=4**
**C4 -** No caso do paciente com dores, que está na fase final de vida, é adequado utilizar um adesivo transdérmico.	**RE=4**
**C5 -** As terapias complementares são importantes para o controle da dor.	**RE=4**
**C6 -** É sempre importante que os membros da família fiquem com o paciente até que ocorra a morte.	**RE=4**
**C7 -** A constipação deve ser aceita como um sintoma secundário, pois a redução da dor é mais importante.	**RE=4**
**C8 -** Os cuidados paliativos requerem apoio emocional constante.	**RE=4**
**C9 -** Na velhice, devido às inúmeras experiências de perda, as pessoas aprenderam a lidar com o luto de forma independente.	**RE=1**
**C10 -** A filosofia dos cuidados paliativos significa que não são mais realizados tratamentos para prolongar a vida.	**RE=4**
**C11 -** Medo e exaustão diminuem a intensidade da dor.	**RE=1**
**C12 -** Pacientes com doenças que ameaçam a vida devem sempre saber da verdade para que possam se preparar para a morte.	**RE=4**
**C13 -** Os membros da equipe não precisam ter fé para poder acompanhar espiritualmente pacientes em fase final de vida.	**RE=4**
**C14 -** Paciente em cuidados paliativos deve aceitar a morte.	**RE=1**
**C15 -** Habilidades de comunicação podem ser aprendidas.	**RE=4**
**C16 -** A morte do paciente não deve ser comunicada para outros pacientes próximos dele e em situação semelhante, para evitar inquietação.	**RE=4**
**C17 -** Os aspectos médicos do tratamento sempre têm prioridade nos cuidados paliativos.	**RE=1**
**C18 -** Rituais visíveis e despedidas quando o paciente morre, devem ser evitados para não causar inquietação.	**RE=1**
**C19 -** O uso de antidepressivos na terapia da dor não faz sentido.	**RE=1**
**C20 -** Ao administrar opioides, os analgésicos adjuvantes não são necessários.	**RE=1**
**C21 -** A fase final compreende os últimos três dias de vida.	**RE=4**
**C22 -** Os sentimentos do cuidador (por exemplo, desprezo) podem ser expressados no acompanhamento.	**RE=4**
**C23 -** As necessidades fisiológicas (por exemplo, sexualidade) ainda são importantes na fase final de vida.	**RE=4**
**AE1 -** Coletar dados objetivos que descrevam o nível de dor do paciente.	
**AE2 -** Orientar o paciente sobre como reduzir a náusea.	
**AE3 -** Informar o paciente e os familiares sobre os cuidados paliativos na instituição.	
**AE4 -** Convencer o médico de família sobre a necessidade dos cuidados paliativos.	
**AE5 -** Reconhecer os problemas atuais do paciente em seu meio social e discuti-los com ele.	
**AE6 -** Planejar um encaminhamento para serviços de cuidados paliativos.	
**AE7 -** Conversar com o paciente ansioso e seus entes queridos para que se sintam seguros.	
**AE8 -** Reconhecer e responder adequadamente às necessidades complexas do paciente em cuidados paliativos.	
**AE9 -** Oferecer ao paciente com dor terapias complementares de relaxamento, adequadas para ele.	
**AE10 -** Conversar com um paciente que expressa o desejo de antecipar a morte.	
**AE11 -** Realizar higiene oral apropriada de pacientes em final de vida.	
**AE12 -** Orientar o paciente sobre os possíveis efeitos colaterais dos medicamentos prescritos.	
**AE13 -** Reconhecer problemas específicos de saúde mental nos pacientes.	
**AE14 -** Integrar aspectos culturais relacionados à morte e o morrer ao cuidar de pacientes em final de vida.	
**AE15 -** Ter empatia e respeitar as condições de vida desconhecidas, dinâmicas familiares e necessidades associadas dos pacientes.	

*Nota: C = conhecimento; AE = autoeficácia; RE = resposta esperada.*

O instrumento demonstrou boa validade de conteúdo (IVC > 0,83), consistência interna mediana para o construto “conhecimento” (alfa de Cronbach = 0,486) e ótima para “autoeficácia” (alfa de Cronbach = 0,852), além de boa confiabilidade geral (ICC > 0,5) e ótima reprodutibilidade (p = 0,046) ^(17)^. Trata-se, portanto, de um instrumento válido e apropriado para uso em estudos no contexto da APS brasileira, contribuindo para a identificação de pontos a serem melhorados na prática clínica em CP, bem como subsidiando reflexões para a qualificação profissional e aprimoramento dos serviços^(17)^.

### Análise de resultados

Como métodos de análise dos resultados, utilizou-se a análise descritiva para caracterizar as informações sociodemográficas e profissionais das variáveis quantitativas. Para as variáveis qualitativas, foi aplicado o teste de igualdade de duas proporções, a fim de comparar frequências entre grupos. O teste de Kruskal-Wallis, uma técnica não paramétrica, foi utilizado para comparar distribuições de uma variável ordinal ou contínua entre três ou mais grupos independentes, especialmente quando os dados não apresentavam distribuição normal. Já a correlação de Spearman foi empregada para avaliar a relação entre duas variáveis ordinais ou quantitativas não paramétricas, permitindo verificar a direção e a força da associação entre elas, mesmo na ausência de linearidade ou normalidade.

Na interpretação dos coeficientes de correlação, foram considerados valores de p ≤ 0,05 indicativos de significância estatística, correlação fraca (valores entre 0 e 0,25), regular (0,25 a 0,50), boa (0,50 a 0,75) e ótima (0,75 a 1,00).

## RESULTADOS

A partir da caracterização sociodemográfica e profissional dos participantes, observou-se que os profissionais tinham entre 24 e 64 anos de idade, sendo a maioria do gênero feminino (83,6%), com 43,8% autodeclarados casados e 36,3% de religião católica. A média de tempo de formação na graduação foi de 12 anos, enquanto o tempo médio de atuação na APS foi de oito anos.

Conforme apresentado na Tabela 1, 45,9% dos participantes atuavam em UBSs. A amostra foi composta por profissionais de diferentes categorias da saúde, sendo 43,8% enfermeiros, 19,2% médicos, e os demais foram classificados como “outros profissionais”, como assistente social (8,9%), farmacêutico (4,8%), fisioterapeuta (5,5%), fonoaudiólogo (0,7%), gerente de unidade de saúde (2,7%), nutricionista (5,5%), odontólogo (6,2%), psicólogo (2,1%) e terapeuta ocupacional (0,7%).

**Tabela 1:** Dados profissionais e de formação acadêmica dos participantes (N = 146)

		n	%	Valor de *p*			n	%	Valor de *p*
**Profissão**	Assistente social	13	8,9%	<0,001	**Tipo de instituição em que fez a graduação**	Privada	114	78,1%	<0,001
	Enfermeiro	64	43,8%	Ref.		Pública	32	21,9%	
	Farmacêutico	7	4,8%	<0,001	**Pós-graduação**	Não possui	13	8,9%	<0,001
	Fisioterapeuta	8	5,5%	<0,001		Possui	133	91,1%	
	Fonoaudiólogo	1	0,7%	<0,001	**Tipo de pós-graduação**	Doutorado	2	1,4%	<0,001
	Gerente	4	2,7%	<0,001		Especialização	129	88,4%	Ref.
	Médico	28	19,2%	<0,001		Mestrado	2	1,4%	<0,001
	Nutricionista	8	5,5%	<0,001		Não tem	13	8,9%	<0,001
	Odontólogo	9	6,2%	<0,001	**Formação** **em CP**	Não possui	129	88,4%	<0,001
	Psicólogo	3	2,1%	<0,001		Possui especialização	17	11,6%	
	Terapeuta ocupacional	1	0,7%	<0,001	**Contato com pacientes** **em CP**	Não possui	38	26,0%	<0,001
						Possui	108	74,0%	
**Tipo de unidade/ equipe que trabalha na APS**	UBS/AMA	8	5,5%	<0,001		NASF	4	2,7%	<0,001
	EMAP	1	0,7%	<0,001		PAI	1	0,7%	<0,001
	EMAD	30	20,5%	<0,001		UBS	67	45,9%	Ref.
	ESF	34	23,3%	<0,001					

*Nota: CP = cuidados paliativos; NASF = Núcleo de Apoio à Saúde da Família; APS = Atenção Primária à Saúde; UBS/AMA = Unidades Básicas de Saúde com Assistência Médica Ambulatorial; EMAP = Equipe Multidisciplinar de Apoio; EMAD = Equipe Multiprofissional de Atenção Domiciliar; ESF = Estratégia Saúde da Família; PAI = Programa de Ações Integradas; UBS = Unidade Básica de Saúde; N = número de participantes; Ref = questão com maior número de respostas.*

**Tabela 2:** Correlação dos fatores quantitativos com as questões do Questionário de Conhecimento e Autoeficácia sobre Cuidados Paliativos (BPW-BR)

	Idade		Tempo de formação		Tempo de serviço		Tempo de atuação na Atenção Primária à Saúde		Tempo de atuação na unidade	
	Corr (r)	Valor de *p*	Corr (r)	Valor de *p*	Corr (r)	Valor de *p*	Corr (r)	Valor de *p*	Corr (r)	Valor de *p*
**C1**	0,131	0,114	0,076	0,364	0,114	0,169	-0,003	0,975	-0,037	0,660
**C2**	-0,125	0,133	-0,034	0,683	-0,061	0,461	-0,168	0,043^*^	-0,050	0,547
**C3**	0,207	0,012^*^	0,196	0,018^*^	0,248	0,003^*^	0,129	0,121	-0,031	0,709
**C4**	0,080	0,335	0,080	0,341	0,143	0,085	0,113	0,175	0,116	0,163
**C5**	0,020	0,806	-0,067	0,423	-0,064	0,446	-0,100	0,230	-0,104	0,211
**C6**	0,094	0,258	0,189	0,023^*^	0,175	0,035^*^	0,063	0,447	-0,029	0,726
**C7**	0,050	0,546	0,146	0,079	0,122	0,142	0,096	0,247	0,027	0,744
**C8**	-0,049	0,559	0,074	0,375	0,040	0,635	0,076	0,361	0,029	0,725
**C9**	0,111	0,182	0,051	0,545	0,117	0,160	0,055	0,511	-0,038	0,649
**C10**	-0,179	0,031^*^	-0,117	0,160	-0,156	0,060	-0,168	0,042^*^	-0,188	0,023^*^
**C11**	0,110	0,188	0,046	0,581	0,025	0,769	0,010	0,909	0,072	0,389
**C12**	0,014	0,869	0,002	0,984	0,020	0,812	-0,049	0,556	0,087	0,298
**C13**	-0,104	0,214	-0,045	0,593	-0,096	0,250	-0,019	0,819	0,066	0,430
**C14**	-0,013	0,878	-0,075	0,367	-0,081	0,329	-0,012	0,884	-0,100	0,229
**C15**	-0,156	0,061	0,005	0,949	-0,035	0,679	0,011	0,899	-0,007	0,932
**C16**	0,181	0,029^*^	0,091	0,275	0,154	0,064	-0,005	0,956	0,047	0,576
**C17**	0,001	0,996	-0,060	0,474	-0,063	0,450	-0,118	0,158	-0,107	0,199
**C18**	0,108	0,194	-0,044	0,603	0,083	0,321	0,072	0,388	0,132	0,113
**C19**	0,123	0,139	0,106	0,203	0,121	0,146	0,060	0,469	-0,088	0,292
**C20**	0,031	0,712	-0,047	0,576	-0,074	0,375	-0,120	0,148	-0,029	0,731
**C21**	0,094	0,261	0,041	0,623	0,044	0,599	-0,006	0,939	-0,029	0,726
**C22**	-0,114	0,171	-0,081	0,331	-0,043	0,606	0,018	0,832	0,113	0,176
**C23**	-0,033	0,695	0,061	0,468	0,008	0,924	0,062	0,458	0,061	0,467
**AE1**	-0,107	0,198	-0,133	0,111	-0,072	0,387	-0,102	0,222	-0,044	0,595
**AE2**	0,073	0,378	-0,006	0,947	0,069	0,407	0,054	0,514	0,050	0,548
**AE3**	0,122	0,141	0,053	0,529	0,002	0,978	0,053	0,523	0,042	0,613
**AE4**	0,026	0,753	0,066	0,432	0,129	0,120	0,198	0,017^*^	0,081	0,334
**AE5**	0,031	0,710	-0,015	0,861	0,063	0,449	0,129	0,120	0,080	0,335
**AE6**	0,125	0,134	-0,028	0,735	0,033	0,691	0,016	0,846	-0,033	0,696
**AE7**	0,056	0,499	-0,044	0,599	0,022	0,795	-0,009	0,914	0,041	0,619
**AE8**	0,042	0,614	-0,035	0,680	0,079	0,346	0,031	0,712	0,046	0,584
**AE9**	0,086	0,304	-0,022	0,793	0,118	0,157	0,087	0,298	0,113	0,174
**AE10**	0,063	0,447	0,007	0,931	0,117	0,158	0,061	0,466	-0,018	0,832
**AE11**	0,160	0,053	0,129	0,121	0,181	0,029^*^	0,240	0,004^*^	0,101	0,226
**AE12**	0,085	0,309	-0,027	0,746	0,072	0,386	0,078	0,350	-0,003	0,973
**AE13**	0,113	0,175	-0,037	0,656	0,085	0,309	0,170	0,040^*^	0,175	0,035^*^
**AE14**	0,061	0,462	-0,014	0,864	0,143	0,084	0,099	0,233	0,025	0,768
**AE15**	0,020	0,808	0,029	0,729	0,108	0,196	0,009	0,916	0,092	0,270

Nota: C = conhecimento; AE = autoeficácia; Corr (r) = correlação;

* = Valor de p ≤ 0,05: _= valor de p próximo a 0,05.

Quanto à formação acadêmica, 78,1% dos participantes cursaram graduação em instituições privadas e 91,1% realizaram cursos de pós-graduação, sendo a maioria em nível de especialização (88,4%). Apesar de 74% relatarem contato prévio com pacientes em CP, apenas 11,6% informaram possuir formação específica na área (Tabela 1).

A Tabela 2 apresenta a análise de correlação entre as respostas ao BPW-BR e idade, tempo de formação, tempo de serviço na saúde, tempo de atuação na APS e tempo de atuação na unidade de trabalho atual.

Nas questões relacionadas ao conhecimento em CP, observaram-se correlações positivas entre: item C2 e tempo de atuação na APS; item C3 e idade, tempo de formação e tempo de serviço; item C6 e tempo de formação e tempo de serviço; item C10 e idade, tempo de atuação na APS e tempo de atuação na unidade; e item C16 e a idade dos participantes. No que se refere à autoeficácia, verificaram-se correlações positivas entre: AE4 e tempo de atuação na APS; AE11 e tempo de serviço e de atuação na APS; e AE13 e tempo de atuação na APS e na unidade de saúde (Tabela 2).

A Tabela 3 apresenta os dados da correlação entre cada uma das respostas do BPW-BR e as variáveis categóricas de gênero, tipo de instituição de graduação, contato prévio com pacientes em CP e formação em CP. O gênero apresentou correlação positiva significativa com os itens C1, C15 e C21, sem correlações observadas com os itens de autoeficácia. A instituição de formação teve correlação positiva com os itens C10, C12, C17 e C20, além dos itens AE3, AE4, AE6, AE7, AE8, AE9, AE10, AE12, AE13 e AE14. O contato prévio com pacientes em CP se correlacionou positivamente com o item C11 e os itens AE3, AE4 e AE5. Não foram observadas correlações entre formação em CP e os itens de conhecimento; no entanto, houve correlação positiva com os itens AE1 e AE4 (Tabela 3).

**Tabela 3:** Correlação dos fatores qualitativos com as questões do Questionário de Conhecimento e Autoeficácia sobre Cuidados Paliativos (BPW-BR)

Gen		M	DP	Valor de *p*	Grad	M	DP	Valor de *p*	Cont CP	M	DP	Valor de *p*	F CP	M	DP	Valor de *p*
C1	F	1,67	1,09	0,029^*^	PR	1,61	1,04	0,786	N	1,68	1,14	0,682	N	1,64	1,00	0,150
	M	1,21	0,72		PB	1,56	1,08		S	1,56	1,02		S	1,29	1,00	
C2	F	1,85	1,11	0,699	PR	1,84	1,08	0,557	N	1,82	1,09	0,907	N	1,81	1,00	0,525
	M	1,67	0,87		PB	1,75	1,08		S	1,82	1,08		S	1,94	2,00	
C3	F	2,62	1,24	0,570	PR	2,72	1,24	0,193	N	2,76	1,26	0,484	N	2,71	3,00	0,109
	M	2,79	1,25		PB	2,41	1,24		S	2,61	1,24		S	2,18	1,00	
C4	F	2,92	1,17	0,437	PR	2,87	1,19	0,128	N	2,97	1,20	0,829	N	2,92	3,00	0,718
	M	3,00	1,35		PB	3,16	1,22		S	2,92	1,20		S	3,00	4,00	
C5	F	3,94	0,23	0,569	PR	3,91	0,34	0,414	N	3,87	0,41	0,193	N	3,93	4,00	0,323
	M	3,83	0,56		PB	3,97	0,18		S	3,94	0,27		S	3,88	4,00	
C6	F	3,39	0,87	0,891	PR	3,44	0,81	0,357	N	3,50	0,69	0,640	N	3,42	4,00	0,405
	M	3,42	0,78		PB	3,25	0,98		S	3,36	0,90		S	3,24	3,00	
C7	F	2,30	1,19	0,073	PR	2,39	1,22	0,791	N	2,26	1,18	0,464	N	2,40	3,00	0,762
	M	2,79	1,18		PB	2,34	1,12		S	2,43	1,21		S	2,29	2,00	
C8	F	3,94	0,23	0,630	PR	3,96	0,21	0,093	N	3,92	0,27	0,607	N	3,95	4,00	0,309
	M	3,92	0,28		PB	3,88	0,34		S	3,94	0,23		S	3,88	4,00	
C9	F	1,79	0,95	0,963	PR	1,76	0,94	0,572	N	1,97	1,00	0,138	N	1,77	1,00	0,627
	M	1,75	0,90		PB	1,84	0,92		S	1,71	0,91		S	1,88	2,00	
C10	F	2,51	1,31	0,347	PR	2,57	1,32	0,038^*^	N	2,71	1,29	0,179	N	2,49	3,00	0,432
	M	2,21	1,35		PB	2,06	1,22		S	2,37	1,32		S	2,24	2,00	
C11	F	1,23	0,67	0,768	PR	1,25	0,69	0,698	N	1,42	0,89	0,049^*^	N	1,25	1,00	0,275
	M	1,21	0,51		PB	1,16	0,45		S	1,16	0,51		S	1,06	1,00	
C12	F	3,07	0,92	0,411	PR	3,17	0,93	0,016^*^	N	3,21	0,87	0,353	N	3,06	3,00	0,389
	M	3,21	0,93		PB	2,81	0,86		S	3,05	0,94		S	3,29	3,00	
C13	F	2,62	1,26	0,360	PR	2,63	1,24	0,376	N	2,79	1,26	0,481	N	2,67	3,00	0,916
	M	2,92	1,10		PB	2,81	1,26		S	2,63	1,23		S	2,65	3,00	
C14	F	2,01	0,97	0,640	PR	2,04	1,00	0,896	N	2,03	0,97	0,975	N	2,00	2,00	0,308
	M	2,13	1,08		PB	2,00	0,95		S	2,03	1,00		S	2,24	2,00	
C15	F	3,95	0,31	0,009^*^	PR	3,91	0,37	0,125	N	3,89	0,51	0,973	N	3,94	4,00	0,233
	M	3,83	0,38		PB	4,00	0,00		S	3,94	0,23		S	3,88	4,00	
C16	F	2,61	1,15	0,407	PR	2,68	1,17	0,343	N	2,89	1,01	0,147	N	2,68	3,00	0,187
	M	2,83	1,09		PB	2,50	1,05		S	2,56	1,18		S	2,35	3,00	
C17	F	2,10	1,12	0,561	PR	2,22	1,17	0,004^*^	N	2,16	1,13	0,574	N	2,12	2,00	0,142
	M	1,96	1,20		PB	1,56	0,84		S	2,05	1,14		S	1,71	1,00	
C18	F	1,55	0,95	0,357	PR	1,66	1,02	0,088	N	1,79	1,07	0,082	N	1,61	1,00	0,256
	M	1,75	1,03		PB	1,31	0,69		S	1,51	0,92		S	1,35	1,00	
C19	F	1,31	0,72	0,440	PR	1,39	0,86	0,573	N	1,29	0,77	0,427	N	1,36	1,00	0,366
	M	1,54	1,06		PB	1,19	0,40		S	1,37	0,79		S	1,24	1,00	
C20	F	1,60	0,96	0,179	PR	1,78	1,10	0,019^*^	N	1,71	1,01	0,484	N	1,70	1,00	0,337
	M	2,04	1,33		PB	1,28	0,68		S	1,66	1,05		S	1,47	1,00	
C21	F	1,61	1,02	0,019^*^	PR	1,79	1,12	0,117	N	1,82	1,14	0,396	N	1,71	1,00	0,923
	M	2,21	1,25		PB	1,44	0,88		S	1,68	1,06		S	1,76	1,00	
C22	F	3,07	1,16	0,415	PR	3,00	1,17	0,319	N	2,89	1,23	0,357	N	2,98	3,00	0,116
	M	2,83	1,31		PB	3,16	1,22		S	3,08	1,17		S	3,41	4,00	
C23	F	3,32	1,04	0,861	PR	3,29	1,05	0,346	N	3,34	1,02	0,975	N	3,32	4,00	0,944
	M	3,42	0,93		PB	3,50	0,92		S	3,33	1,02		S	3,47	4,00	
AE1	F	3,61	0,80	0,474	PR	3,61	0,78	0,523	N	3,61	0,68	0,565	N	3,54	4,00	0,009^*^
	M	3,54	0,78		PB	3,53	0,84		S	3,59	0,83		S	4,00	4,00	
AE2	F	3,49	0,89	0,617	PR	3,51	0,91	0,441	N	3,45	1,06	0,774	N	3,46	4,00	0,083
	M	3,54	0,93		PB	3,47	0,84		S	3,52	0,84		S	3,82	4,00	
AE3	F	3,63	0,72	0,923	PR	3,72	0,68	<0,001^*^	N	3,32	1,02	0,024^*^	N	3,60	4,00	0,201
	M	3,54	0,93		PB	3,25	0,88		S	3,72	0,61		S	3,76	4,00	
AE4	F	3,41	0,94	0,485	PR	3,29	1,02	0,020^*^	N	2,97	1,20	0,007^*^	N	3,30	4,00	0,005^*^
	M	3,21	1,14		PB	3,69	0,74		S	3,52	0,85		S	3,94	4,00	
AE5	F	3,63	0,68	0,580	PR	3,66	0,64	0,334	N	3,34	0,94	0,018^*^	N	3,64	4,00	0,469
	M	3,54	0,78		PB	3,47	0,88		S	3,71	0,56		S	3,47	4,00	
AE6	F	3,43	0,84	0,092	PR	3,55	0,77	0,033^*^	N	3,32	0,99	0,284	N	3,44	4,00	0,265
	M	3,63	0,88		PB	3,16	1,05		S	3,52	0,79		S	3,65	4,00	
AE7	F	3,74	0,56	0,623	PR	3,82	0,50	<0,001^*^	N	3,55	0,80	0,080	N	3,73	4,00	0,318
	M	3,79	0,51		PB	3,47	0,62		S	3,81	0,41		S	3,88	4,00	
AE8	F	3,31	0,85	0,249	PR	3,39	0,87	<0,001^*^	N	3,16	0,95	0,391	N	3,22	3,00	0,064
	M	3,04	1,04		PB	2,84	0,85		S	3,31	0,87		S	3,59	4,00	
AE9	F	3,26	0,99	0,524	PR	3,33	1,01	0,003^*^	N	3,05	1,11	0,215	N	3,23	4,00	0,718
	M	3,08	1,18		PB	2,88	0,98		S	3,30	0,98		S	3,24	4,00	
AE10	F	3,11	1,03	0,117	PR	3,32	0,93	0,001^*^	N	2,97	1,22	0,422	N	3,13	3,00	0,174
	M	3,46	0,88		PB	2,66	1,12		S	3,24	0,93		S	3,47	4,00	
AE11	F	3,34	1,06	0,348	PR	3,46	1,00	0,056	N	3,50	0,86	0,688	N	3,40	4,00	0,434
	M	3,58	0,83		PB	3,13	1,10		S	3,34	1,08		S	3,24	4,00	
AE12	F	3,43	0,92	0,582	PR	3,53	0,87	0,017^*^	N	3,39	0,97	0,830	N	3,43	4,00	0,374
	M	3,54	0,83		PB	3,19	0,97		S	3,47	0,88		S	3,59	4,00	
AE13	F	3,49	0,78	0,519	PR	3,61	0,72	0,001^*^	N	3,32	0,96	0,176	N	3,49	4,00	0,753
	M	3,58	0,78		PB	3,16	0,88		S	3,57	0,70		S	3,65	4,00	
AE14	F	3,52	0,77	0,395	PR	3,55	0,80	0,011^*^	N	3,24	1,00	0,077	N	3,47	4,00	0,863
	M	3,29	1,04		PB	3,22	0,87		S	3,56	0,74		S	3,53	4,00	
AE15	F	3,94	0,23	0,630	PR	3,96	0,21	0,093	N	3,92	0,27	0,607	N	3,93	4,00	0,263
	M	3,92	0,28		PB	3,88	0,34		S	3,94	0,23		S	4,00	4,00	

Nota: C = conhecimento; AE = autoeficácia; M = média; DP = Desvio Padrão; Grad = instituição de graduação; Cont CP = contato com pacientes em cuidados paliativos; F = feminino; M = masculino; PR = privada; PB = pública; N = não; S = sim; CP = formação em cuidados paliativos;

* = Valor de p ≤ 0,05; _= valor de p próximo a 0,05.

**Tabela 4:** Comparação entre as respostas dadas pelos participantes de acordo com a profissão

	Profissão	Média	Desvio Padrão	Valor de *p*		Profissão	Média	Desvio Padrão	Valor de *p*
**CONHECIMENTO**									
**C1**	Enfermeiro	1,75	1,18	0,183	**C2**	Enfermeiro	1,98	1,12	0,013^*^
	Médico	1,29	0,76			Médico	1,29	0,60	
	Outros	1,57	0,98			Outros	1,91	1,14	
**C3**	Enfermeiro	2,78	1,29	0,319	**C4**	Enfermeiro	2,91	1,16	0,012^*^
	Médico	2,46	1,10			Médico	3,46	0,96	
	Outros	2,59	1,25			Outros	2,69	1,27	
**C5**	Enfermeiro	3,97	0,18	0,371	**C6**	Enfermeiro	3,53	0,71	0,046^*^
	Médico	3,89	0,42			Médico	2,96	1,14	
	Outros	3,89	0,37			Outros	3,46	0,77	
**C7**	Enfermeiro	2,42	1,18	0,023^*^	**C8**	Enfermeiro	3,98	0,13	0,122
	Médico	1,86	1,04			Médico	3,89	0,31	
	Outros	2,61	1,23			Outros	3,91	0,29	
**C9**	Enfermeiro	1,75	0,94	0,720	**C10**	Enfermeiro	2,55	1,31	0,809
	Médico	1,68	0,82			Médico	2,39	1,34	
	Outros	1,87	0,99			Outros	2,39	1,32	
**C11**	Enfermeiro	1,25	0,64	0,464	**C12**	Enfermeiro	3,08	0,93	0,464
	Médico	1,11	0,42			Médico	2,93	0,98	
	Outros	1,26	0,73			Outros	3,19	0,89	
**C13**	Enfermeiro	2,41	1,29	0,075	**C14**	Enfermeiro	1,95	0,93	0,537
	Médico	2,86	1,15			Médico	1,96	1,07	
	Outros	2,89	1,18			Outros	2,15	1,02	
**C15**	Enfermeiro	3,88	0,45	0,147	**C16**	Enfermeiro	2,95	1,09	0,002^*^
	Médico	4,00	0,00			Médico	2,07	1,09	
	Outros	3,96	0,19			Outros	2,57	1,13	
**C17**	Enfermeiro	2,19	1,13	0,032^*^	**C18**	Enfermeiro	1,70	1,05	0,073
	Médico	1,57	0,92			Médico	1,18	0,48	
	Outros	2,20	1,19			Outros	1,65	1,01	
**C19**	Enfermeiro	1,41	0,90	0,990	**C20**	Enfermeiro	1,89	1,13	0,005^*^
	Médico	1,25	0,52			Médico	1,18	0,61	
	Outros	1,33	0,75			Outros	1,67	1,03	
**C21**	Enfermeiro	1,78	1,12	0,682	**C22**	Enfermeiro	2,97	1,15	0,059
	Médico	1,61	1,07			Médico	3,43	1,10	
	Outros	1,69	1,04			Outros	2,91	1,23	
**C23**	Enfermeiro	3,30	1,05	0,090					
	Médico	3,71	0,60						
	Outros	3,19	1,12						
**AUTOCONHECIMENTO**									
**AE1**	Enfermeiro	3,72	0,63	0,032^*^	**AE2**	Enfermeiro	3,69	0,66	0,004^*^
	Médico	3,75	0,65			Médico	3,79	0,50	
	Outros	3,37	0,98			Outros	3,13	1,15	
**AE3**	Enfermeiro	3,73	0,60	0,297	**AE4**	Enfermeiro	3,47	0,85	<0,001^*^
	Médico	3,39	0,99			Médico	3,89	0,31	
	Outros	3,59	0,77			Outros	3,00	1,18	
**AE5**	Enfermeiro	3,61	0,70	0,596	**AE6**	Enfermeiro	3,52	0,80	0,393
	Médico	3,71	0,66			Médico	3,29	0,94	
	Outros	3,57	0,72			Outros	3,50	0,86	
**AE7**	Enfermeiro	3,80	0,57	0,266	**AE8**	Enfermeiro	3,48	0,76	0,024^*^
	Médico	3,71	0,46			Médico	2,96	1,04	
	Outros	3,70	0,57			Outros	3,17	0,91	
**AE9**	Enfermeiro	3,53	0,78	0,009^*^	**AE10**	Enfermeiro	3,25	0,94	0,320
	Médico	2,96	0,96			Médico	3,00	0,94	
	Outros	3,02	1,21			Outros	3,17	1,13	
**AE11**	Enfermeiro	3,78	0,58	<0,001^*^	**AE12**	Enfermeiro	3,70	0,68	0,004^*^
	Médico	3,14	1,11			Médico	3,50	0,69	
	Outros	3,04	1,23			Outros	3,13	1,12	
**AE13**	Enfermeiro	3,63	0,72	0,179	**AE14**	Enfermeiro	3,58	0,75	0,375
	Médico	3,43	0,79			Médico	3,43	0,74	
	Outros	3,41	0,84			Outros	3,39	0,94	
**AE15**	Enfermeiro	3,95	0,21	0,530					
	Médico	3,89	0,31						
	Outros	3,94	0,23						

Nota: C = conhecimento; AE = autoeficácia;

* = Valor de p ≤ 0,05; _= valor de p próximo a 0,05.

Para avaliar possíveis diferenças nas respostas ao BPW-BR entre as categorias profissionais, realizou-se análise comparativa, descrita na Tabela 4. Os participantes foram agrupados em três categorias, como “enfermagem”, “medicina” e “outros profissionais”, de modo a garantir proporcionalidade suficiente para análise estatística. Verificaram-se diferenças expressivas entre os grupos nas questões C2, C4, C6, C7, C16, C17 e C20, relacionadas ao conhecimento, e AE1, AE2, AE4, AE8, AE9, AE11 e AE12, relacionadas à autoeficácia (Tabela 4).

## DISCUSSÃO

Compreender o que a equipe multiprofissional da APS conhece sobre CP, bem como as crenças sobre sua aptidão no cuidado a esse perfil de pacientes, é de suma importância para o planejamento de treinamentos sobre o tema e para o desenvolvimento e aprimoramento de políticas públicas, como a PNCP. Tal compreensão pode contribuir para a oferta de um cuidado antecipado e de maior qualidade às pessoas em necessidade de CP nos serviços de porta de entrada e de promoção da saúde, como propõe a APS^(4)^.

A aprovação da PNCP representa um avanço significativo, uma vez que permitirá a destinação de recursos financeiros e educacionais específicos para ações que promovam os CP na APS^(7,18)^, reforçando a relevância de estudos como este, que subsidiam estratégias educacionais ao identificarem necessidades práticas e de aprendizagem dos profissionais, possibilitando o direcionamento e a otimização dos recursos.

A maioria dos participantes deste estudo (74%) relatou ter contato com pacientes em CP, apesar de 88,4% não possuírem formação específica sobre o tema (Tabela 1). Embora a literatura sobre a inserção dos CP na APS ainda seja escassa, há consenso de que a ausência de conhecimento formal sobre CP pode dificultar a identificação precoce de casos elegíveis, especialmente nos contextos em que não há malignidade envolvida ou entre pacientes mais jovens^(19)^. Com a implementação da PNCP, espera-se a criação de novos cursos de formação sobre CP voltados aos profissionais da APS, além da adoção de algoritmos, instrumentos e estratégias para o reconhecimento precoce dos pacientes elegíveis, bem como maior clareza sobre os fluxos de atendimento na RAS^(7,18)^.

Entre os achados deste estudo, observou-se que os profissionais mais velhos e com maior tempo de formação e de atuação demonstraram mais conhecimento sobre a administração medicamentosa, a importância da presença familiar no fim da vida e a filosofia dos CP. Aqueles com mais tempo de atuação na APS apresentaram melhor desempenho na autoeficácia para comunicação com o médico sobre a necessidade de CP, na realização de higiene oral adequada no fim da vida e no reconhecimento de problemas de saúde mental nos pacientes (Tabela 2). Esses resultados corroboram a literatura, que aponta que profissionais mais experientes costumam se sentir mais seguros e confiantes em suas atribuições, quando comparados aos mais jovens^(20)^. Outros estudos também demonstram que maior tempo de atuação na APS está diretamente relacionado a maior conhecimento sobre CP^(9,21)^. Contar com pessoas mais experientes nos treinamentos pode ser uma experiência interessante, além de proporcionar uma troca de conhecimento intergeracional.

Profissionais da APS que relataram contato prévio com pacientes em CP apresentaram melhor compreensão sobre a influência das emoções na intensidade da dor e maior autoeficácia na comunicação com pacientes, familiares e equipe médica (Tabela 3). Uma comunicação eficaz entre todos os envolvidos nos CP favorece condutas mais assertivas, proporcionando conforto e viabilizando a elaboração de planos de ação eficazes. Apesar de desafiadora, sobretudo para os médicos, a comunicação é uma habilidade essencial que pode e deve ser desenvolvida, configurando-se como ponto chave em treinamentos voltados à atuação em CP na APS^(22)^.

Diferentemente de estudos internacionais^(9,21,22)^, não foi observada correlação entre o conhecimento sobre CP e o fato de o profissional ter recebido formação prévia na área. No entanto, identificou-se correlação entre autoeficácia e coleta de dados sobre dor e comunicação com médicos sobre a prescrição de CP. Embora a literatura aborde extensivamente o conhecimento em CP, pouco se discute sobre o papel da autoeficácia nas boas práticas assistenciais. Sabe-se que maior conhecimento promove maior autoeficácia, e esta, por sua vez, pode contribuir para a oferta de cuidados de melhor qualidade^(11,23,24)^. Dessa forma, recomenda-se que as capacitações incluam, além do conteúdo teórico, ações práticas e de planejamento^(12)^, por meio de estágios presenciais e discussões de casos clínicos, para que os profissionais se sintam mais seguros na prática clínica.

A análise das respostas dos participantes segundo o tipo de instituição de formação revelou que aqueles formados em instituições privadas (78%) apresentaram melhor conhecimento sobre a filosofia dos CP e comunicação do prognóstico ao paciente. Também obtiveram pontuações mais elevadas nas questões relacionadas à autoeficácia em comunicação com pacientes e familiares, gestão do cuidado, e oferta de terapias complementares. Já os profissionais formados em instituições públicas demonstraram maior conhecimento sobre a atuação em equipe nos CP e administração de opioides, além de se sentirem mais preparados para dialogar com o médico de família sobre a necessidade de CP (Tabela 3).

A inclusão dos CP na formação dos profissionais de saúde ainda é recente. No Brasil, a partir de 2022, tornou-se obrigatória a oferta de disciplina específica sobre CP nos cursos de medicina^(25)^. Estudo com 2.225 médicos brasileiros recém-formados apontou que 99,2% consideram importante a inclusão de CP na graduação; contudo, apenas 46,2% relataram ter recebido formação no tema, sendo que os oriundos de instituições privadas relataram maior exposição a treinamentos em CP do que os formados em instituições públicas (57% *versus* 51%)^(26)^. Esses dados evidenciam a relevância da formação em CP ainda durante a graduação. Nos demais cursos da área da saúde, a obrigatoriedade dessa formação ainda não está em vigor, mas constitui um passo essencial que poderá mudar a percepção que os demais profissionais têm sobre seu papel nos CP, além de agregar muito ao cuidado dos pacientes, que precisam essencialmente de cuidados multidisciplinares.

Ao comparar as categorias profissionais, os médicos apresentaram maior conhecimento e autoeficácia quanto ao manejo farmacológico da dor e coleta de dados sobre dor, incluindo a prescrição de opioides, em consonância com estudos realizados na Espanha, Vietnã e Malásia^(9,10,27)^. No entanto, tais estudos apontam que esse conhecimento é mais evidente entre profissionais com experiência prévia em hospitais ou serviços especializados em CP, além de evidenciar a restrição ao acesso a medicamentos como a morfina, realidade semelhante à brasileira^(28)^. Importante destacar que, no Brasil, apenas médicos estão autorizados a prescrever opioides, o que pode justificar o maior conhecimento entre esses profissionais^(28)^.

Os médicos também relataram maior autoeficácia em orientar sobre o manejo de náuseas e convencer o médico de família quanto à necessidade dos CP. Todavia, como já identificado na literatura, demonstraram menor desempenho na identificação de necessidades complexas dos pacientes e na valorização da presença familiar no processo de finitude (Tabela 4). Revisão sistemática australiana apontou que, embora reconheçam a importância da comunicação no fim da vida, os médicos se sentem despreparados e mal treinados, encontrando dificuldades estruturais para tal, inclusive com falhas na comunicação entre os membros da equipe, o que repercute negativamente na interação com pacientes e familiares^(29)^. Dado que a comunicação não é uma habilidade inata, destaca-se a importância de que seja ensinada, praticada e constantemente aprimorada, visando à qualificação do cuidado em CP.

Foi demonstrada dificuldade na oferta de terapias complementares de relaxamento e de conhecimento limitado sobre a constipação como sintoma secundário por parte dos médicos (Tabela 4). Em revisão sistemática que incluiu dados do Brasil, observou-se que, apesar de os médicos da APS serem os mais abertos às práticas integrativas e complementares, sua aceitação gira em torno de 65%^(30)^. Ainda que haja evidências positivas sobre práticas como acupuntura, reflexologia e massagem em CP^(31)^, muitos médicos mostram pouco interesse em se especializar nelas, delegando sua aplicação a outros membros da equipe.

Os enfermeiros, de forma geral, apresentaram os melhores indicadores de autoeficácia diante das condutas em CP, especialmente na identificação das necessidades dos pacientes, oferta de terapias complementares, higiene oral e orientação sobre efeitos colaterais de medicamentos. Demonstraram também melhor desempenho na comunicação com pacientes e familiares, embora tenham apresentado menor conhecimento sobre uso de adesivos transdérmicos e prescrição de opioides (Tabela 4). A literatura aponta que, embora o conhecimento teórico dos enfermeiros sobre os princípios dos CP ainda necessite de fortalecimento, suas condutas e atitudes no cuidado ao paciente terminal são um diferencial^(9,13,21)^, o que corrobora os achados deste estudo.

O papel do enfermeiro na atenção à saúde, especialmente nos CP, está fortemente relacionado ao gerenciamento de casos, à comunicação com familiares, ao suporte contínuo e ao manejo da dor por estratégias farmacológicas e não farmacológicas^(32)^. Assim, é fundamental que as capacitações ampliem os fundamentos teóricos sobre CP, abordando, inclusive, seus conceitos e princípios, pontos a serem melhorados na prática, já identificadas em diversos estudos nacionais e internacionais ^(9,11-13,21,23)^, bem como neste trabalho. A enfermagem pode ter um papel importante no matriciamento do restante da equipe na APS, já que conta com mais experiência nesses que são pontos chave para os CP.

Os demais profissionais de saúde, que não médicos ou enfermeiros, tiveram desempenho significativamente inferior em várias questões de conhecimento e, sobretudo, de autoeficácia, destacando-se a dificuldade autorrelatada em sugerir ao médico a prescrição de CP. Juntamente com os enfermeiros, também apresentaram piores resultados na questão C17, que aborda a priorização de aspectos médicos em detrimento de outras intervenções, indicando que os próprios profissionais da equipe multidisciplinar não conseguem reconhecer e valorizar suas contribuições na equipe (Tabela 4).

A *American Society of Clinical Oncology* reconhece que os CP devem ser prestados por equipes interprofissionais, citando profissionais como assistentes sociais, fisioterapeutas, fonoaudiólogos, nutricionistas, psicólogos e terapeutas ocupacionais, todos incluídos neste estudo. No entanto, o *guideline* limita-se a menções breves, sem apresentar evidências específicas sobre cada área profissional, sendo suas recomendações mais voltadas às dimensões físicas e psicológicas^(31)^. Estudos com psicólogos em CP são mais frequentes e indicam que sua atuação deve abranger tanto o paciente e sua família quanto o apoio à equipe interprofissional^(33)^. Embora também haja estudos sobre fisioterapeutas^(34)^ e assistentes sociais^(35)^ em CP, inexistem pesquisas específicas sobre sua atuação na APS, o que reforça a necessidade de investigar essas categorias mais profundamente, a fim de subsidiar o desenvolvimento de instrumentos e capacitações adequados. Por isso, este estudo escolheu o BPW-BR, que foi validado no contexto brasileiro e com diversos profissionais^(17)^.

Os CP, conceitualmente, exigem uma abordagem em equipe^(36)^. Contudo, na APS, há dificuldades na integração efetiva da equipe, na comunicação entre profissionais e na troca de saberes. Além disso, a própria configuração da APS no Brasil resulta em uma menor presença desses profissionais, limitando sua participação em estudos^(4,8)^. No presente estudo, tivemos dificuldade em aprofundar as análises em cada categoria separadamente, justamente porque há um número reduzido desses profissionais nas equipes de APS.

Apesar de a promoção da interprofissionalidade ser um princípio da APS, observa-se uma atuação ainda predominantemente unilateral^(37)^. A falta de conhecimento sobre CP por parte dos profissionais também compromete o reconhecimento das demandas dos pacientes e seus familiares, dificultando a indicação de condutas e a argumentação técnica sobre CP junto à equipe^(38)^. Portanto, são necessários estudos com amostras mais representativas dessas categorias para verificar se o BPW-BR, em seu formato atual, é adequado para avaliar conhecimento e autoeficácia da equipe interprofissional da APS em CP e verificar, de forma mais específica, quais os pontos a serem melhorados em cada categoria profissional.

Os achados deste estudo corroboram as evidências da literatura quanto aos pontos a serem melhorados na formação em CP para todos os profissionais de saúde que atuam na APS. Capacitações específicas nessa área são fortemente recomendadas, tanto para promover o domínio teórico e filosófico dos CP quanto para desenvolver habilidades em comunicação, manejo da dor (farmacológico e não farmacológico) e promoção da interprofissionalidade no cuidado.

### Limitações do estudo

Como fatores limitantes da pesquisa, podemos considerar o número reduzido de participantes, que se deu especialmente pela alta demanda dos profissionais de saúde envolvidos no atendimento de casos de COVID-19 ou até mesmo infectados pelo vírus no período de coleta^(39)^. Além disso, há a baixa variabilidade regional de atuação dos profissionais, já que todos trabalham na cidade de São Paulo, impossibilitando extrapolar esses resultados para as demais regiões do Brasil sem que outros estudos sejam feitos.

O número reduzido de profissionais inclusos na categoria “outros” impossibilitou a análise de dados com maior distinção entre cada uma das profissões, mas recomendamos fortemente que mais pesquisas sejam feitas com essas categorias profissionais, inclusive para entender se o BPW-BR é um bom instrumento para analisar seu conhecimento e autoeficácia sobre CP.

É importante ter clareza sobre a consistência interna do construto “conhecimento” na validação do BPW-BR, cujo alfa de Cronbach foi de 0,486^(17)^, valor considerado mediano^(40)^, sugerindo a importância de validação do BPW-BR com um número maior de participantes, de diferentes regiões do Brasil, para verificar se há problemas estruturais de consistência interna nos itens sobre conhecimento ou se foi um valor enviesado pelo número amostral.

### Contribuições para a área da saúde

Como contribuições para a área da saúde, foi possível obter um melhor entendimento sobre o que os profissionais da APS sabem e se sentem hábeis e capazes para realizar nos CP. Por meio dessas informações, é possível desenhar, de forma mais objetiva, programas de capacitação específicos para as demandas de profissionais da APS. O estudo foi pioneiro ao trazer informações sobre outros profissionais de saúde que não só médicos e enfermeiros, deixando claro que é necessário expandir pesquisas com outras categoriais profissionais que são fundamentais na prestação dos CP e precisam ser parte ativa das decisões e intervenções.

Como possíveis estratégias para educar os profissionais sobre CP na APS, sugerimos que o assunto seja pauta em reuniões de matriciamento e de programas de educação continuada, e que se aproveite a implementação da PNCP para pleitear treinamentos, especializações e verba para melhoria da infraestrutura e contratação de pessoal. Mais educação sobre o assunto também auxiliará na identificação de pacientes elegíveis aos CP, melhorando a qualidade de vida e levando mais informações para a população assistida.

## CONCLUSÕES

A partir dos dados sociodemográficos e dos resultados do BPW-BR, foi possível traçar um panorama do conhecimento e da autoeficácia em CP entre os diferentes profissionais que compõem as equipes da APS. De forma geral, todas as categorias apresentaram conhecimento e autoeficácia insatisfatórios sobre o tema.

Os pontos a serem melhorados na prática clínica foram as habilidades de comunicação entre equipes e com pacientes e familiares, manejo da dor, conhecimento técnico e conceitual dos CP e entendimento do papel de cada profissional da saúde dentro dos CP na APS.

## Data Availability

Os dados de pesquisa disponíveis estão no corpo do artigo.
